# Does Whipping Tournament Incentives Spur CSR Performance? An Empirical Evidence From Chinese Sub-national Institutional Contingencies

**DOI:** 10.3389/fpsyg.2022.841163

**Published:** 2022-02-25

**Authors:** Muhammad Kaleem Khan, Shahid Ali, R. M. Ammar Zahid, Chunhui Huo, Mian Sajid Nazir

**Affiliations:** ^1^Asia-Australia Business College, Liaoning University, Shenyang, China; ^2^School of Management Science and Engineering, Nanjing University of Information Science and Technology, Nanjing, China; ^3^School of Accounting, Yunnan Technology and Business University, Kunming, China; ^4^Institute of Administrative Sciences, University of the Punjab, Lahore, Pakistan; ^5^HEC Montreal, University of Montreal, Montreal, QC, Canada

**Keywords:** corporate social performance, CEO tournament incentives, sub-national institutional contingencies, tournament theory, foreign ownership, development

## Abstract

The current study investigates whether tournament incentives motivate chief executive officer(s) (CEOs) to be socially responsible. Furthermore, it explores the role of sub-national institutional contingencies [i.e., state-owned enterprises (SOE) vs. non-SOEs, foreign-owned entities (FOE) vs. non-FOEs, cross-listed vs. non-cross-listed, developed region] in CEO tournament incentives and the corporate social responsibility performance (CSRP) relationship. Data were collected from all A-shared companies listed in the stock exchanges of China from 2014 to 2019. The study uses the baseline methodology of ordinary least squares (OLS) and cluster OLS regression. Moreover, firm-fixed effects regression, two-stage least squares regression, and propensity score matching deal with the endogeneity problem and check the robustness of the results. The results provide reliable evidence that tournament incentives motivate CEOs to be more socially responsible. On the other hand, sub-national institutional contingencies positively affect the association between CEO tournament incentives and CSRP. The findings have important implications for companies and regulators who wish to enhance CSP by providing incentives to top managers.

## Introduction

Earlier researchers have extensively considered the various corporate social responsibility (CSR) perspectives and their effects on the economy (Jo and Harjoto, 2011). Later, corporate finance literature focused on the financial and accounting determinants of a CSR performance (CSRP) such as government, the external stakeholders' significance (David et al., 2007), society (Matten and Moon, 2008), institutional pressure (Matten and Moon, 2008; Bondy et al., 2012), etc. Others contributed to advancing the relevant strand of literature by investigating internal factors, including the chief executive officer's (CEO's) political ideology, the ethical commitment of the top management team (Muller and Kolk, 2010), CEO overconfidence (McCarthy et al., 2017), the CEO's power (Jiraporn and Chintrakarn, 2013; Li et al., 2016), gender diversity in the boardroom (McGuinness et al., 2017), executive compensation (Jian and Lee, 2015), and the firm's financial condition. Last but not least, the top management role cannot be overlooked in determining the firm's ethical and social orientation because of the concentration of decision-making power (Waldman et al., 2006).

Currently, researchers have started explaining the ‘competition' as another important determinant of CSR (Zhao et al., 2021). The idea of competition has its roots in the economic theory, namely in tournament theory, which describes variances in managerial performance compensation (Lazear and Rosen, 1981; O'Reilly et al., 1988). The theory of the tournaments applies to a contest in which managers are eligible for bonuses and other benefits. The pay gap between CEO and other executives leads to good competition among managers, leading to better business results. The executives are motivated by substantial incentives for the winner of tournaments. This gap between the winner of the competition and the person in second place can be an operational incentive mechanism as the executives are evaluated on relative instead of absolute performance. Therefore, we expect that increasing the incentives/gap among executives and CEOs can make them more socially responsible for proving as deserving of that prize. According to tournament theory, Lazear and Rosen (1981) propose that pay disparity can be explained as prizes paid to contestants in the labor market according to their rank order.

Institutional contingencies are diverse characteristics of institutions within the same economy. He and Fang (2016) named these institutional discrepancies as sub-national institutional contingencies. This study has researched the most vital aspect of contingencies in the sub-national context for Chinese firms. We established an empirical endeavor for different patterns, such as listed companies' patterns (non-cross-listed and cross-listed), ownership patterns (non-state and state ownership), and regional patterns (less-developed and developed regions).

This research revolves around the intersection of three concepts: CSRP, tournament incentives, and subnational institutions. The study aims to explore the relationship between CSRP and tournament incentives and CSRP and subnational institutions. Moreover, the study projects the moderating role of subnational institutions in bridging CSRP and tournament incentives. The purpose and finding of the study add valuation contribution in CSR literature and tournament theory. The study aims to acquire insight into the mechanism through which the CSRP is motivated by the incentive scheme. This study has been built upon the notion that incentives motivate executives to take actions that can have financial and social implications. Incentivized CEOs face a loss if they demonstrate undesirable CSRP, as the market reacts strongly to it, resulting in a decrease in market value (McGuire et al., 2019). Conversely, robust CSRP is considered the only insurance choice whose unknown advantages are visible in performance deficiencies (Cassimon et al., 2016). Incentive plans can encourage managers to be socially responsible, mitigating agency conflicts (Cai et al., 2011). Executives are more willing to take higher risks to win a tournament, resulting in more effective operating, financial, and social policies (Goel and Thakor, 2008).

When we consider the case in the context of China, it takes on further significance. China is an important emerging economy with a high degree of economic growth, providing more ownership structure diversity (Khan et al., 2017) in many sub-national areas (Chan et al., 2010). In contrast, some fresh studies have found that the Chinese economy has already emerged, so it no longer possesses the emerging economy title. For example, Bruton et al. (2021) and Zhang et al. (2021) have convincingly argued that China has emerged as an aspirant economy. How tournament incentives affect the CSRP in a freshly emerged economy could be an interesting story to tell. Nevertheless, China has many firms listed across regions such as Hong Kong, London, and New York (He and Fang, 2016; Khan et al., 2021). This institutional variation enables us to investigate its impact on CSRP.

Even though China's economy is still heavily regulated by the government, the country's central planning system has been gradually replaced by more market-oriented policies. Promotion within the CCP/Government hierarchy with lifelong benefits, such as job security, housing subsidies, pensions, and medical treatment, is important for executives working in government-controlled companies. As a result, we expect that tournament cash incentives in Chinese companies will be weaker than in Western companies. As a first step in making tournament cash incentives weaker, there is a strong non-cash incentive (political promotion). Another factor that makes tournament prizes less appealing is that CEO pay in publicly traded companies controlled by the government is typically capped at multiples (between 3 and 15 times) of the average worker's wage (Firth et al., 2006). Culturally, China has a high level of collectivism, which includes a greater emphasis on equality (Hofstede, 2001). As a socialist country, China focuses on promoting social harmony. A “reasonable,” but not excessive, pay disparity between managerial levels is therefore expected by the general public.

Second, we extend the literature beyond developed countries by providing the first empirical study from China's largest developing country to the best of the authors' knowledge. China's institutional factors are unique (Guariglia and Yang, 2016; Ali et al., 2019). Scholars have recommended that the most promising corporate governance focuses on understanding the institutional factors in which governance occurs (Davis, 2005). We extend the existing literature on the relationship between government ownership and CSRP (Fan et al., 2007; Li et al., 2016; Khan et al., 2019) by exploring for the first time whether the effect of CEO tournament incentives on CSRP varies between state-owned enterprises (SOE) and non-SOEs (NSOEs). We find that the positive effect of CEO tournament incentives on CSRP is more pronounced in SOEs than in NSOEs. These results suggest that SOEs can benefit in the context of CSRP from providing high tournament incentives to their CEOs.

Previously available researchers have demonstrated that executives with high CSRP tend to receive larger pay packages; it makes the CEOs involved in corporate social responsibility (CSR) (Krüger, 2015). However, these studies do not provide a comprehensive picture since they only recognize the CEO's overall compensation rather than CEO tournament incentives, i.e., the pay differential between executives and CEOs. The present study contributes to the existing literature on the internal drivers of CSRP by investigating CEOs' tournament incentives as determinants of CSRP for the first time. The study fills another gap. Compared with the existing literature, present research adds following other significant contributions. First, this research contributes to the literature on CSRP in-house drivers by examining CEOs' tournament incentives as determinants of CSRP for the first time. Second, this study explores whether diverse sub-national institutes have a different connection with a company's CSRP. This work proposes that sub-national institutes are active in compelling and smoothing premeditated selections. Our findings suggest that managers are driven by bonuses and prizes (in line with tournament theory) in the Chinese market. Growth-inducing salary rewards allow executives to compete with one another, allowing the company to flourish financially and socially. Further, this study's outcomes reveal sub-national institutional contingencies [i.e., firms in less-developed vs. more-developed regions, non-cross-listed vs. cross-listed companies, non-foreign-owned entities (FOEs) vs. FOEs, non-SOEs vs. SOEs] positively affect CSRP. The fallouts of this research divulge that CSRP in firms in more-develop areas, cross-listed companies, FOEs, and SOEs are higher than their counterparts.

Section Theoretical Discussions and Relevant Work will discuss the theoretical foundation and associated literature in detail. Data and Research methodology details are in section Data and Methodology, followed by discussions of results and conclusions, respectively, in sections Results and Conclusions and Policy Implementations.

## Theoretical Discussions and Relevant Work

### Role of Top Executives in Taking CSR

Top management is responsible for key decision-making. A firm's corporate social responsibility performance indicates the orientation and priorities of the company's executives. In this context, the agency theory also supported the role of top management for corporate environmental and social performance disclosure practices (Jensen and Meckling, 1976; Cordeiro et al., 2013). According to another theory, known as Upper-Echelon theory, managers (particularly CEOs) play a critical role in the selection and implementation of strategic decisions that ultimately affect the performance or growth of a company (Hambrick and Mason, 1984; Hambrick, 2007); this includes decisions related to corporate social responsibility (CSR) initiatives. According to this perspective, these characteristics (e.g., age, functional tracks, career experiences, and education) of top executives are important determinants of the strategic decisions made by firms in relation to social practices. Several studies have found that the characteristics of top management (CEOs in particular) can encourage greater executives' commitment to compliance with institutional regulations, which can have a positive impact on environmental sustainability and performance (Ntim and Soobaroyen, 2013; Zahid and Simga-Mugan, 2019; Grofčíková, 2020; Lu et al., 2020; Malkawi and Khayrullina, 2021).

### Tournament Theory

The pay gap between the CEO and the next level is typically quite large (Gomez-Mejia, 1994), and managerial marginal product arguments (O'Reilly et al., 1988) do not provide a convincing explanation for this phenomenon. According to tournament theory, which states that workers in the labor market compete for rewards based on their position in the competition, Lazear and Rosen (1981) propose that this discrepancy can be explained. They argue that the competition for CEO positions could be likened to a tournament, where the prizes are fixed in advance and participants put forth an effort to increase their chances of winning a prize that isn't based on one's absolute performance but rather on one's performance relative to other competitors (Conyon et al., 2001). It is argued by Rosen (1986) that large top prizes are theoretically required for tournament survivors to be motivated so that they do not rest on their past achievements when they enter the final contest. When monitoring costs are high, contests make sense to determine compensation packages. According to tournament theory, executive pay should have a convex relationship with the organizational level. The prize (gap) and the number of participants should also have a positive relationship. Finally, the company's performance should be positively correlated with executive wage dispersion (O'Reilly et al., 1988).

### CEO Tournament Incentives and CSRP

It is still unclear how tournament rewards impact firm CSRP. Some studies have approached this problem in a roundabout way, but the precise relationship remains unknown. We begin by describing the literature on the association between tournament rewards and firm performance, keeping in mind that CSR is directly linked to firm performance (Ali et al., 2019). Second, we discuss the literature stream that advocates that compensation (the main player in tournament theory) is directly linked with CSP. Last, we discuss the scarce literature which links tournament incentives with CSRP directly or indirectly.

Some evidence suggests that CSR reward helps to affect CSRP (Hong et al., 2016). Based on tournament theory, the CEO's payout seems to be better than what you might expect (Vo and Canil, 2019). The CEO typically gets higher pay because of their additional duties that stem from its overall success (O'Reilly et al., 1988). Hannan et al. (2008) claimed that CEOs compete for performance when prizes are awarded according to ability. Rivalry breeds executive pay inequality in an organization (Gnyawali et al., 2008). CEOs focus on CSR activities due to innate enjoyment of incentives, which is directly linked to the success of the company, as well as extrinsic motivations; hence the organizations' goals and values are related (Petrenko et al., 2016). CEOs may be compensated for their CSR-related nonfinancial benefits, such as satisfying shareholders, increasing the company's image and promoting respect, and the cause of social responsibility. Where executives believe their image can be advanced by working on corporate social responsibility, they're inclined to spend their time and money on CSR to achieve it (Barnea and Rubin, 2010).

Chief executive officer (CEO) remuneration, especially incentives, may directly affect decisions concerning CSR deeds. Monitoring mechanisms by the board of directors can help CEOs' incentives align with shareholders' concerns, which according to the job match theory, is a good match between the CEO and firm reflected by better firm performance. The prevailing view is that businesses try to incentivize CSR activities because CSR is an essential component of their long-term, lasting sustainability and viability. Competitive compensation for executives can cause internal conflict and lead to less involvement in corporate social responsibility, making resolution difficult (Cai et al., 2011). Also, CEOs' compensation appears to be reduced in companies that attract a lot of media coverage. They are thusly bombarded with shareholder demands, potentially resulting in less greed. According to Mr. Potts, CEOs who have high ethical and social responsibility earn lower pay than those who do not as a result. There can incite violent out burgeoning conduct in senior management, including excessive risk-taking and risk-taking for personal gain (Becker and Huselid, 1992). An important consideration when planning a tournament is that it rewards people based on their output; as a result, it provides an effective motivation to improve, increasing overall production (Connelly et al., 2014). Similarly, research has found that salary inequality serves as valid evidence of corporate success (Lazear and Rosen, 1981; Hu et al., 2013; Elkins, 2018; Elsayed and Elbardan, 2018). One notable problem with this literature is that it does not account for corporate social responsibility with tournament theory.

The literature on tournaments and CSR growth is still incented. Nothing on this subject has been concluded, although some scholars have tried to investigate it. CEO personality and CSR motivations are intertwined (Petrenko et al., 2016). Over-investing in “corporate social responsibility” for social media acceptance and appreciation (Galaskiewicz, 1997; Barnea and Rubin, 2010). Due to the interests of CEOs directing their money toward the organization's clients, staff, and vendors, these stakeholders are more likely to help the firm's day-to-to-day activities. CEOs are compensated because their companies have higher ROI (Jian and Lee, 2015; Ali et al., 2019). The prize money will make the scheme work better and have more value (Kini and Williams, 2012). The importance of a formal system of corporate governance for compensation of CSR influences CSR (Hong et al., 2016). We have yet to establish a definitive connection between tournament pay and playing well. Tournament rewards have been unable to define precisely the influence of CSSR participation. Furthermore, no research on this question has been conducted in China.

Therefore, tournament theory encourages productivity. Competitors perform better if the compensation is provided with regard to the tournament view to winning in mind (Hannan et al., 2008). Tournament theory points out a pay disparity between executives, leading to increased hostility among colleagues, strengthening competition, leading to better company results as those executives invest in C-E. In agreement with conventional wisdom, we believe that CEO tournament rewards will inspire them to spend more on corporate social responsibility because the prestige of the company and their goods will be enhanced, the credibility of the CEO will be strengthened, and trust will be restored among stakeholders. The studies have overwhelmingly shown that managers do their best work when motivated by bonuses, such as rewards and prize money.

Conventional wisdom states that companies should reward CEOs for sustainable growth with CSR. Since prize money is given based on contrast, we believe that CEOs and executives will enhance the gap between them instead of reducing it. Often, CEOs tend to work for the firm's financial success as well as CSR. Since these CEOs will receive tournament rewards, it follows that there is a positive relationship between CSRP and tournament performance.

*H*_1_*: CEOs' tournament incentive is positively associated with CSRP*.

### Role of Sub-national Institutional Contingencies in CEO Tournament Incentives and CSP Nexus

#### CEO Tournament Incentives and CSRP in SOEs vs. Non-SOEs

China's SOEs have two main goals: managing products or services markets. Second, the state restricts SOEs from engaging in CSR to gain political support. As a result, SOE executives are interested in the government's priorities and strategies in order to gain rewards like tournaments or promotional incentives (Xu et al., 2015). Due to political ties and government constraints, CEOs of SOEs are more likely to improve the organization's image by CSR (Marquis and Qian, 2014) than CEOs from non-SOEs (Zheng and Zhang, 2016).

Furthermore, SOE executives are often selected and promoted through political maneuvering. As a result, SOE executives and board members are encouraged to make decisions in the government's best interests, prioritizing social goals over financial benefits (Firth et al., 2007). Public criticism of executive pay and CEO success also increases for SOEs (Hu et al., 2013). Government agencies track CEO results since the state offers sufficient financial support to SOEs (Musacchio et al., 2015). This motivates executives to participate in politically approved CSR (Campbell, 2007). The Chinese government also provides incentives to SOE executives (Hung et al., 2012) in exchange for the company's participation in CSR (Li et al., 2016).

*H*_2_*: CEO tournament incentives' incremental impact on CSRP is more keenly recognized in SOEs than non-SOEs firms*.

#### CEO Tournament Incentives and CSRP in FOEs vs. Non-FOEs

Prior literature revealed that foreign ownership raises (Firth et al., 2007). Thus, FOEs have higher pay-performance sensitivity. The literature indicates that foreign ownership affects firms' outcomes such as strategic investment (David et al., 2006), performance (Yoshikawa et al., 2010), wage (Yoshikawa et al., 2005), redundancies, and adoption of global governance codes (Yoshikawa et al., 2010).

Firms' CSR performance increases with the degree of internalization in China (Cheung et al., 2015). There is underlying evidence that global counterparties, particularly from developed markets, possessed more ingrained and enduring attitudes concerning CSR. Executives' decision-making can be steered in a specific direction through a compensation structure (Bebchuk et al., 2002). Traditionally, the goal was to encourage executives to maximize profits by aligning their interests with shareholders (Kini and Williams, 2012). Nevertheless, if firms' goals are to encourage executives to further environmental and social objectives, their incentives may be used to align with CSR. Foreign owners pressurize corporations to pay for performance systems to incentivize CEOs (Firth et al., 2007). We contend that executive compensation incentivizes top executives to make financial, social, and strategic decisions and that FOEs influence these actions. Given the discussions above, we expect the following result.

*H*_3_*: CEO tournament incentives' incremental impact on CSRP is more keenly accepted in FOEs than non-FOEs firms*.

#### CEO Tournament Incentives and CSRP in Cross-Listed vs. Non-cross-Listed Firms

Cross-listing condenses barriers and offers access to the capital market and group of investors. Cross-listed companies achieve higher market performance than their counterpart (Doidge et al., 2004), higher-earning announcements and abnormal returns (Del Bosco and Misani, 2016), external financing (Reese Jr and Weisbach, 2002) more excellent analyst coverage (Lang et al., 2003) low information asymmetry, squat cost of capital (Hail and Leuz, 2009). Moreover, cross-listed firms also gain extraordinary transaction volumes in domestic markets (Smith and Sofianos, 1997). Furthermore, internationalization provides opportunities for leveraging and learning to understand from diverse institutional settings (i.e., foreign and domestic markets). In China, firms cross-listed in HKSE also cross-listed on the London Stock Exchange or New York Stock Exchange (He and Fang, 2016).

Diverse business environments and governance systems (Matten and Moon, 2008) imply that companies will encounter best practices, local needs, and social priorities besides cross-listing expectations that differ from domestic markets (Del Bosco and Misani, 2016). When investigating the United Kingdom (UK) and Canadian organizations listed in the United States (US) markets, Southam and Sapp (2010) witness that cross-listing is associated with an increase in executives' pay. Cross-listed organizations pay greater rewards for their executives than domestic organizations. Chinese companies choose to cross-list on the stock market of Hong Kong, where executives enjoy a significant pay gap due to western-designed institutional lucidity and greater inequality tolerance because they are more concerned about shareholder value than social equity. Consequently, Chinese cross-listed firms incorporate more incentives in their pay design (Berrone and Gomez-Mejia, 2009). Furthermore, increase sends to increase after cross-listing (Boubakri et al., 2016). Consequently, cross-listing encourages firm executives to boost CSRP by improving governance through adhering to foreign regulations and norms, increasing the reputation of a company to enhance its plea to stakeholders and investors, overcome foreignness liability, enhance competitiveness (Jo and Harjoto, 2011), and mitigate litigation risk and regulatory burden (Boubakri et al., 2016). These benefits suggest higher CSRP in cross-listed firms.

*H*_4_*: CEO tournament incentives' incremental impact on CSRP is more keenly accepted in cross-listed firms than non-cross-listed ones*.

#### CEO Tournament Incentives and CSRP in More-Developed Region vs. Less-Developed Region Firms

The unique Chinese institutional context encompasses differences in governance mechanisms according to the firm's regional location (more-developed or less-developed region). The market development level in Chinese regions differs significantly from each other. The factor and commodity markets are highly developed, and the legal framework and market intermediaries are similar to those in developed economies (Shi et al., 2012). Contrastingly, organizations in less-developed regions are less strict at law enforcement, have poor local government effectiveness, more intervention, and more exploitation (Chan et al., 2010). The literature indicates that firms' internal monitoring quality is affected by regions. In executives' compensation, pay-performance and turnover-performance sensitivity are weak due to low external monitoring in an organization in under-developing regions (Conyon and He, 2014).

In China, local governments are free to make policies to develop market intermediaries, factor markets, and product markets. Thus, strategic choices vary in a regional environment (Chan et al., 2010). Market reforms have caused significant progress, but huge gaps still exist among less-developed and developed regions (Fan et al., 2003). In China, different regions of the country have a difference in institutional mechanisms and market development (Fan et al., 2007). Developed regions are associated with a more formal structure, better protection of investors' interests, stronger governance mechanisms, and improved civil rights protection (Cordeiro et al., 2013).

Moreover, some scholars have established that a firm's internal monitoring and control also differ in the context of regional contingencies. For instance, Conyon and He (2011) studied executive compensation and corporate governance links in Ch. They found that the CEO pay-performance link is more keenly felt in more-developed regions than in less-developed regions. Another study reported a weaker CEO compensation-performance nexus in the framework of less developed areas (Firth et al., 2007).

Recently, due to rising environmental issues, such as air contamination, water disposal, and greenhouse gas emissions, both the public and government have grown demand for accountability and sustainability progress (Zheng et al., 2014). Therefore, in more-developed regions, executives chasing tournament incentives are more likely to affect CSP than expected in less-developed areas for two reasons. One may increase legality among stakeholders and the society, while the other may be to gain subsidies and incentives associated with going green. Therefore, to develop an indulgence of whether the impact of CEO tournament incentives on CSP prevails in the same way in both developed and less-developed regions, we categorize our sample firms into more-developed regions and less-developed regions. Taken together, we make the following predictions:

H_5_: *CEO tournament incentives' incremental impact on CSRP is more keenly accepted in the more-developed region*.

## Data and Methodology

### Data Description

The current study employs the China Stock Market and Accounting Research (CSMAR) for data collection. CSP data was gained from Ranking (RKS), which delivers sovereign standing for listed corporations in China (Wu et al., 2016). From 2014 to 2019, the first sample includes A and H share corporations listed on the Hong Kong, Shanghai, and Shenzhen stock exchanges. For reliable results, missing values were not included, and the final sample involved 11,991 observations. To avoid extreme values, we winsorize continuous variables at the 1st and 99 percentiles. We first calculated other executives' CEO and average compensation and merged this data set with CSR rating data. Then, we merged the data of all control variables used in multiple analyses and deleted 3,838 firm-year observations with missing data needed for the firm-level control variables and 9,276 firm-year observations with a missing value for the CEO-level control variables. The final tournament incentive-CSP sample is 12,881 firm-year observations. To test the hypothesis with sub-national institutional contingencies, we merge the data with sub-national institutional contingencies (i.e., state vs. non-State organizations, foreign-owned vs. non-foreign-owned, cross-listed companies vs. non-cross-listed companies, more-developed region companies vs. less-developed region's company's) data. This procedure leads to a final sample of 11,991 firm-year observations for the study.

### Variable Measurements

#### Dependent Variable

Corporate Social Responsibility Performance (CSRP): Consistent with previous literature, we use RKS's social ratings based on the GRI 3 reformed to the Chinese perspective (Lau et al., 2016; McGuinness et al., 2017). RKS determines social ratings for three principal areas of reporting, such as Macrocosm (Overall), Content, and Technique. CSR reports encompass three main dimensions (overall evaluation, content evaluation, and technical evaluation) further subdivided into 70 sub-dimensions of CSR activities. The overall dimension, which is further subdivided into 14 sub-dimensions, assesses social responsibility policy, stakeholder engagement, and knowledge comparability among the other dimensions.

#### Independent Variables

Chief executive officer (CEO) tournament incentive: following prior studies (Chen et al., 2011; Kini and Williams, 2012; Hu et al., 2013; Vo and Canil, 2019), the primary variable of interest is CEOs' tournament incentive. We measure Tournament_Incentives as the difference in compensation between CEOs and other executives. First, we calculate the average executive pay by dividing the total compensation paid to the executives by the total number of executives. Second, we calculate the CEO pay gap by dividing the total CEO compensation by the average compensation paid to executives. Finally, we used two measures to quantify the tournament reward to ensure robust results: CEO_PayGap, measured as the logarithm of CEOs' total pay minus the average compensation of executives, and CEO_PayGapRatio, measured as the ratio of CEO pay to executives' compensation (Chan et al., 2010; Hu et al., 2013; He and Fang, 2016). The following equations present the measurement:


(1) $$\begin{array}{l} CEO\text{\_}PayGap=Log\left(CEOPay-Ave.ExecutivePay\right) \end{array}$$

(2) $$\begin{array}{l} CEO\text{\_}PayGapRatio=\left(\frac{CEO\;Pay}{Ave.ExecutivePay}\right) \end{array}$$

#### Moderating Variables (Sub-national Institutional Contingencies)

State-owned enterprises (SOEs vs. non-SOEs): In line with previous literature (Conyon and He, 2014; He and Fang, 2016), the SOE is set for 1 if the government or state is the owner 0 otherwise.

Foreign ownership (FOEs vs. non-FOEs): Following McGuinness et al. (2017), FOE is 1 for foreign-owned enterprises and 0 otherwise.

Cross-listing (cross-listed vs. non-cross-listed firms): We defined cross-listed firms (Cross_Listed), the cross-listed firm is coded as 1 if the firm is listed in Hong Kong stock exchange, and 0 otherwise, as measured in prior studies (He and Fang, 2016).

Regional development (more-developed-region vs. less-developed-region): In line with previous studies (Cordeiro et al., 2013; He and Fang, 2016), we defined the developed region (D_Region) as the dummy variable which equals to 1 if a firm's head office is listed in the more developed region of China, and 0 otherwise (for further details of variables see the Table A1 in Supplementary Material).

### Empirical Models

To test our entire hypothesis following models are estimated. The first is to test the effect of CEO tournament incentives on CSRP (Equations 3 and 4). Second, we test how SNIC moderates CEO tournament incentives and the CSRP nexus (Equations 5–8). Following previous studies (Barnea and Rubin, 2010; McGuinness et al., 2017; Fernández-Gago et al., 2018; Ali et al., 2019), we use ordinary least squares (OLS) and cluster OLS regression to test equations. The following are the equations of the study:


(3) $$\begin{array}{l} \text{CSRP}{\;}_{\text{it}}=\;\alpha +\;\beta_{1}{\text{CEO\_PayGap}}_{\text{it}}+\beta_{2}{B\text{\_}Size}_{\text{it}}\\ \;+\beta_{3}{\text{B\_Ind}}_{\text{it}}+\beta_{4}{\text{B\_Share}}_{\text{it}}+\;\beta_{5}{\text{B\_FemaleP}}_{\text{it}}\\ \;+\beta_{6}{\text{CEO\_Duality}}_{\text{it}}+\beta_{7}{\text{CEO\_Tenure}}_{\text{it}}\\ \;+\beta_{8}{\text{CEO\_Degree}}_{\text{it}}+\beta_{9}\text{SO}{\text{E}}_{\text{it}}+\beta_{10}\text{FO}{\text{E}}_{\text{it}}\\ \;+\beta_{11}{\text{F\_Size}}_{\text{it}}+\beta_{12}{\text{F\_Age}}_{\text{it}}+\beta_{13}{\text{F\_GrowOpp}}_{\text{it}}\\ \;+{{\text{β}}_{14}TobinQ}_{it}+\;\beta_{15}{F\text{\_}Growth}_{\text{it}}+\beta_{16}{\text{F\_Leverage}}_{\text{it}}\\ \;+\;\beta_{17}{\text{Cross\_Listed}}_{\text{it}}+\beta_{18}{\text{D\_Region}}_{\text{it}}\\ \;+\overset{n}{\underset{i=1}{\sum }}\beta_{n}{\text{Industry\_Dummies}}_{\text{it}}\\ \;+\overset{n}{\underset{i=1}{\sum }}\beta_{n}{\text{Year\_Dummies}}_{\text{it}}\varepsilon_{\text{it}} \end{array}$$

(4) $$\begin{array}{l} \text{CSRP}{\;}_{\text{it}}=\;\alpha +\;\beta_{1}{\text{CEO\_PayGapRatio}}_{\text{it}}+\;\beta_{2}{B\text{\_}Size}_{\text{it}}\\ \;+\beta_{3}{\text{B\_Ind}}_{\text{it}}+\beta_{4}{\text{B\_Share}}_{\text{it}}\\ \;+\;\beta_{5}{\text{B\_FemaleP}}_{\text{it}}+\beta_{6}{\text{CEO\_Duality}}_{\text{it}}\\ \;+\beta_{7}{\text{CEO\_Tenure}}_{\text{it}}+\beta_{8}{\text{CEO\_Degree }}_{\text{it}}\\ \;+\beta_{9}\text{SO}{\text{E}}_{\text{it}}+\beta_{10}\text{FO}{\text{E}}_{\text{it}}+\beta_{11}{\text{F\_Size}}_{\text{it}}+\beta_{12}{\text{F\_Age}}_{\text{it}}\\ \;+\beta_{13}{\text{F\_GrowOpp}}_{\text{it}}+\beta_{14}{\text{TobinQ}}_{\text{it}}+\;\beta_{15}{F\text{\_}Growth}_{\text{it}}\\ \;+\beta_{16}{\text{F\_Leverage}}_{\text{it}}+\;\beta_{17}{\text{Cross\_Listed}}_{\text{it}}\\ \;+\beta_{18}{\text{D\_Region}}_{\text{it}}+\overset{n}{\underset{i=1}{\sum }}\beta_{n}{\text{Industry\_Dummies}}_{\text{it}}\\ \;+\overset{n}{\underset{i=1}{\sum }}\beta_{n}{\text{Year\_Dummies}}_{\text{it}}\varepsilon_{\text{it}} \end{array}$$

(5) $$\begin{array}{l} \text{CSRP}{\;}_{\text{it}}=\;\alpha +\;\beta_{1}{\text{CEO\_PayGap}}_{it}{+\;\beta }_{2}\text{SO}{\text{E}}_{\text{it}}\\ \;+\;\beta_{3}{\text{CEO\_PayGap}}_{it}\times {\text{SOE}}_{\text{it}}\\ \;{+\;\beta }_{4}{B\text{\_}Size}_{\text{it}}+\beta_{5}{\text{B\_Ind}}_{\text{it}}+\beta_{6}{\text{B\_Share}}_{\text{it}}\\ \;+\beta_{7}{\text{B\_FemaleP}}_{\text{it}}+\beta_{8}{\text{CEO\_Duality}}_{\text{it}}\\ \;+\beta_{9}{\text{CEO\_Tenure}}_{\text{it}}+\beta_{10}{\text{CEO\_Degree }}_{\text{it}}\\ \;+\beta_{11}\text{FO}{\text{E}}_{\text{it}}+\beta_{12}{\text{F\_Size}}_{\text{it}}+\beta_{13}{\text{F\_Age}}_{\text{it}}\\ \;+\beta_{14}{\text{F\_GrowOpp}}_{\text{it}}+{{\text{β}}_{15}TobinQ}_{\text{it}}\\ \;+\;\beta_{16}{\text{F\_Growth}}_{\text{it}}+\beta_{17}{\text{F\_Leverage}}_{\text{it}}\\ \;+\;\beta_{18}{\text{Cross\_Listed}}_{\text{it}}+\beta_{19}{\text{D\_Region}}_{\text{it}}\\ \;+\overset{n}{\underset{i=1}{\sum }}\beta_{\text{n}}{\text{Industry\_Dummies}}_{\text{it}}\\ \;+\overset{n}{\underset{i=1}{\sum }}\beta_{\text{n}}{\text{Year\_Dummies}}_{it}\varepsilon_{\text{it}} \end{array}$$

(6) $$\begin{array}{l} \text{CSRP}{\;}_{\text{it}}=\;\alpha +\;\beta_{1}{\text{CEO\_PayGap}}_{\text{it}}{+\;\beta }_{2}\text{FO}{\text{E}}_{\text{it}}\\ \;+\;\beta_{3}{\text{CEO\_PayGap}}_{\text{it}}\times {\text{FOE}}_{\text{it}}{+\;\beta }_{4}{B\text{\_}Size}_{\text{it}}\\ \;+\beta_{5}{\text{B\_Ind}}_{\text{it}}+\beta_{6}{\text{B\_Share}}_{\text{it}}+\;\beta_{7}{\text{B\_FemaleP}}_{\text{it}}\\ \;+\beta_{8}{\text{CEO\_Duality}}_{\text{it}}+\beta_{9}{\text{CEO\_Tenure}}_{\text{it}}\\ \;+\beta_{10}{\text{CEO\_Degree }}_{\text{it}}+\beta_{11}\text{SO}{\text{E}}_{\text{it}}+\beta_{12}{\text{F\_Size}}_{\text{it}}\\ \;+\beta_{13}{\text{F\_Age}}_{\text{it}}+\beta_{14}{\text{F\_GrowOpp}}_{\text{it}}\\ \;+{{\text{β}}_{15}TobinQ}_{\text{it}}+\;\beta_{16}{\text{F\_Growth}}_{\text{it}}+\beta_{17}{\text{F\_Leverage}}_{\text{it}}\\ \;+\;\beta_{18}{\text{Cross\_Listed}}_{\text{it}}+\beta_{19}{\text{D\_Region}}_{\text{it}}\\ \;+\overset{n}{\underset{i=1}{\sum }}\beta_{\text{n}}{\text{Industry\_Dummies}}_{\text{it}}\\ \;+\overset{n}{\underset{i=1}{\sum }}\beta_{\text{n}}{\text{Year\_Dummies}}_{\text{it}}\varepsilon_{\text{it}} \end{array}$$

(7) $$\begin{array}{l} \text{CSRP}{\;}_{\text{it}}=\;\alpha +\;\beta_{1}{\text{CEO\_PayGap}}_{\text{it}}{+\;\beta }_{2}{\text{Cross\_Listed}}_{\text{it}}\\ \;+\;\beta_{3}{\text{CEO\_PayGap}}_{\text{it}}\times {\text{Cross\_Listed}}_{\text{it}}{+\;\beta }_{4}{\text{B\_Size}}_{\text{it}}\\ \;+\beta_{5}{\text{B\_Ind}}_{it}+\beta_{6}{\text{B\_Share}}_{\text{it}}+\;\beta_{7}{\text{B\_FemaleP}}_{\text{it}}\\ \;+\beta_{8}{\text{CEO\_Duality}}_{\text{it}}+\beta_{9}{\text{CEO\_Tenure}}_{\text{it}}\\ \;+\beta_{10}{\text{CEO\_Degree }}_{\text{it}}+\beta_{11}\text{SO}{\text{E}}_{\text{it}}+\beta_{12}\text{FO}{\text{E}}_{\text{it}}\\ \;+\beta_{13}{\text{F\_Size}}_{\text{it}}+\beta_{14}{\text{F\_Age}}_{\text{it}}+\beta_{15}{\text{F\_GrowOpp}}_{\text{it}}\\ \;+{{\text{β}}_{16}TobinQ}_{\text{it}}+\;\beta_{17}{\text{F\_Growth}}_{\text{it}}\\ \;+\beta_{18}{\text{F\_Leverage}}_{\text{it}}+\beta_{19}{\text{D\_Region}}_{\text{it}}\\ \;+\overset{\text{n}}{\underset{i=1}{\sum }}\beta_{\text{n}}{\text{Industry\_Dummies}}_{\text{it}}\\ \;+\overset{n}{\underset{i=1}{\sum }}\beta_{n}{\text{Year\_Dummies}}_{\text{it}}\varepsilon_{\text{it}} \end{array}$$

(8) $$\begin{array}{l} \text{CSRP}{\;}_{\text{it}}=\;\alpha +\;\beta_{1}{\text{CEO\_PayGap}}_{\text{it}}{+\;\beta }_{2}{\text{D\_Region}}_{\text{it}}\\ \;+\;\beta_{3}{\text{CEO\_PayGap}}_{\text{it}}\times {\text{D\_Region}}_{\text{it}}{+\;\beta }_{4}{B\text{\_}Size}_{\text{it}}\\ \;+\beta_{5}{\text{B\_Ind}}_{\text{it}}+\beta_{6}{\text{B\_Share}}_{\text{it}}+\;\beta_{7}{\text{B\_FemaleP}}_{\text{it}}\\ \;+\beta_{8}{\text{CEO\_Duality}}_{\text{it}}+\beta_{9}{\text{CEO\_Tenure}}_{\text{it}}\\ \;+\beta_{10}{\text{CEO\_Degree }}_{\text{it}}+\beta_{11}\text{SO}{\text{E}}_{\text{it}}+\beta_{12}\text{FO}{\text{E}}_{\text{it}}\\ \;+\beta_{13}{\text{F\_Size}}_{it}+\beta_{14}{\text{F\_Age}}_{it}+\beta_{15}{\text{F\_GrowOpp}}_{\text{it}}\\ \;+{{\text{β}}_{16}TobinQ}_{\text{it}}+\;\beta_{17}{\text{F\_Growth}}_{\text{it}}+\beta_{18}{\text{F\_Leverage}}_{\text{it}}\\ \;+\;\beta_{19}{\text{Cross\_Listed}}_{\text{it}}\\ \;+\overset{n}{\underset{i=1}{\sum }}\beta_{n}{\text{Industry\_Dummies}}_{\text{it}}\\ \;+\overset{n}{\underset{i=1}{\sum }}\beta_{n}{\text{Year\_Dummies}}_{\text{it}}\varepsilon_{\text{it}} \end{array}$$

where the subscript i indicates the firms and t indicate the years throughout the analysis. We include the year and two-digital code industry dummies to avoid any common trend in CSRP. CSRP refers to corporate social responsibility performance (i.e., CSR_Rating) defined as weighted average rating score apportioned by Rankins (RKS) ranging from 0 to 100; CEO_Pay Gap refers to the pay gap between executives and CEO, which defined total compensation of a CEO minus average compensation of all other executives; CEO_Pay Gap Ratio refers to the ratio between CEO and executives' compensation (defined as ratio between CEO and executives' average compensation); CEO_Pay Gap × SOE refers to interaction effect of SOE in CEO Tournament incentives and CSP; CEO_Pay Gap × FOE refers to interaction effect of FOE in CEO Tournament incentives and CSP; CEO_Pay Gap × Cross_Listed refers to interaction effect of Cross_Listed in CEO Tournament incentives and CSP; CEO_Pay Gap × D_Region refers to interaction effect of D_Region in CEO Tournament incentives and CSP; B_Size refers to board size (defined as total number board directors); B_Ind refers as board independence (defined as the proportion of outside directors on the board); B_Share refers as board share (defined as the proportion of shares held by board directors); B_FemaleP refers to portion of female directors (defined percentage of female board directors); CEO_Duality refers to CEO duality (well-defined as if the CEO has a dual role as Chairperson then dummy variable equals 1, and 0 otherwise); CEO_Tenure refers as CEO tenure (as the total number of years since the CEO joined as CEO in a firm); CEO_Degree refers to CEO degree education (equals 1 if the CEO has at least a bachelor degree, and 0 otherwise); SOE refers to state-owned enterprises (defined as a dummy variable, which equals 1 if the local or central government is the dominant owner, and 0 otherwise); FOE refers to foreign owned enterprises (defined as a dummy variable that equals 1 if the foreign investors owned shares in a firm, and 0 otherwise); F_Size, F_Age, F_GrowOpp, TobinQ, F_Growth, F_Leverage, Cross_Listed, D_Region indicates corporation size, company age, organization development chances, Tobin's Q ratio, company growth, organization leverage, cross-listed companies and advance region corporations, respectively. Total sales are used as the corporation size in log form. Age is the number of years listed. The book-to-market ratio is the organization's development chance. The variation in company assets is used as company growth. Debt to asset ratios is used as organizational leverage. Finally, cross-listed companies and advanced region corporations are dummy variables of this study; Industry_Dummies refer to industry effect on CSRP; Year_Dummies refers to year dummies to control the year effect on CSRP.

## Results

### Descriptive Statistics and Correlation Analysis

Table A2 in Supplementary Material shows an increasing trend in CSRP in the sample period, which shows that Chinese companies are showing more intentions toward CSRP along with financial performance. The CEO's average compensation trend in China is also increasing. The proportion of independent directors increases gradually over the period, which shows an improvement in corporate governance in China. Similarly, board room gender diversity also increases another prediction of strong corporate governance in China. This decrease in SOEs shows that Chinese listed firms transition toward the Anglo-Saxon model. Other essential variables, such as CEO duality and CEO tenure, show an increasing trend. Firm age is increasing with the time that Chinese listed firms are performing well to continue their business for a more extended period. Most of the Chinese firms are located in the more developed region of China, and the number of companies is growing in the developed area.

Chief executive officer (CEO) pay gap has a mean of RMB 407,000, indicating that CEOs earn an average of RMB 407,000 more per year than other executives. Another metric of tournament incentive (CEO PayGapRatio) has a mean of 2.59 and a *SD* of 1.2, indicating that CEOs are paid 2.59 times as much as other executives. According to CSR Rating, the average compound CSR rating for Chinese companies is 27.61%, with a maximum score of 89.29 in China. The average board size in China is 10.26, with an average of 38% independent directors, which meets the CSRS requirement that independent directors make up one-third of all listed companies' board members. The average SOE is 46%, and the trend in China is decreasing as a result of the reform program (Khan et al., 2019). On average, CEO tenure is 3.44% in China, with a 2.93% *SD*. The board's average proportion of shares apprehended is 10%, with a *SD* of 0.18.

In China, 24% of CEOs have a double role as CEO and chairman, with a *SD* of 42%. The mean value of the CEO degree is 93%, with a *SD* of 25%. The average FOEs in China are just 5%, with a 23% *SD*. The mean firm size is 21.98 and 1.25 *SD*, and the maximum organization size is 28. The average age is 9.1 years, with a *SD* of 6.22. The maximum age of the firm is 28 years in China. The mean firm growth opportunity was 0.98, and 0.99 *SD* and the average market performance of the listed Chinese company is 2.76, with a *SD* of 0.08. The average firm growth in a sample period is 0.83, with a minimum of −12.81 and a maximum growth of 64.7%. The mean value of firm financial leverage is 0.45, with a 0.36 *SD*. Table A3 in Supplementary Material shows that 6% of the studied companies are cross-listed on different stock markets, especially in Hong Kong and 64%of firms have their head office in the more developed region of China.

The average CSRP of the Chinese sub-national institutional contingencies is portrayed in Table A3 in Supplementary Material. The average CSRP of non-SOEs is 26.78, while the social performance regarding SOEs is 32.77. The *SD*s are 16.25 and 21.22, respectively. The results reveal that SOEs are inclined to contribute more to social deeds. Similarly, the CSR performance of firms with foreign owners is higher than that of firms with no foreign owners. The mean value of CSRP in FOEs is 35.65, with a *SD* of 21.4, while the mean value of non-FOEs is 28.85, with a *SD* of 18.53. The cross-listed in Hong Kong or other stock exchanges have a mean value of 33.38, and non-cross-listed firms have a 25.1 mean CSRP value. The average CSRP of cross-listed companies is 33.38, while for non-cross-listed companies, it is 25.1. The average CSRP in the developed region companies is 30.34, and the maximum is 90.25. The firm is located in the more-developed region frontrunner as equated with firms in the less-developed area.

The correlation between CEO tournament incentives and CSRP is consistent with our prediction, suggesting that CEO tournament incentives motivate CEOs to be more socially responsible. The correlation between Tobin Q and CSRP is 0.03, which specifies the confirmatory association between CSRP and CFP, consistent with our hypothesis. The correlation coefficient between the B_Female P and CEO_Pay Gap is also negative, which indicates that high B_Female P advances the detachment of the compensation committee and limits CEOs' undue compensation.

The correlation (Table A4 in Supplementary Material) between CSRP and SOEs is 0.16; the results predict the positive association between SOEs firms and CSRP, which is consistent with our conjecture in H2, suggesting that the firm's CSRP is higher in SOEs firms. The correlation coefficient of foreign-owned firms 0.11 indicates a positive association between FOEs and CSRP, consistent with H3. A correlation coefficient is positively significant between cross-listed companies and CSRP. Likewise, the same relationship is predicted for D_Region and CSRP, which validates the H4 and H5 of the study. However, the correlation coefficient between sub-national institutional contingencies (i.e., firms in less-developed vs. more-developed regions, non-cross-listed vs. cross-listed companies, non-FOEs vs. FOEs, non-SOEs vs. SOEs) is positive and significant with CEO_Pay Gap, which is consistent with our prediction, suggesting that CEOs receive incentives for being socially responsible. We estimated the regression separately for each tournament incentive measure to alleviate multicollinearity.

### CSRP and Tournament Incentives

The CEOs' tournament bonuses, according to H1, are positively related to CSRP. Table 1 shows the results of two statistical models (OLS and Cluster OLS) for the relationship between CEO tournament incentives and CSP and the regression results of CEO tournament incentives and CSRP. Columns I and II contain the OLS regression results, while columns III and IV contain the cluster-OLS results. To account for cross-sectional dependency in the residuals, T-statistics in III and IV are considered on standard errors company clustered and shown in parentheses.

**Table 1:** Effect of CEO tournament incentive on corporate social responsibility performance.

	**OLS**		**Cluster-OLS**	
	**I**	**II**	**III**	**IV**
CEO_PayGap	2.76*** (13.75)	–	2.76*** (9.07)	–
CEO_PayGapRatio	–	0.71*** (4.88)	–	0.70*** (3.36)
B_Size	−0.16** (−2.15)	−0.15** (−2.35)	−0.17* (−1.95)	−0.15* (−1.76)
B_Ind	2.28* (1.74)	3.24*** (2.79)	2.28* (1.69)	3.23** (2.08)
B_Share	3.52*** (2.94)	3.02*** (2.54)	3.51*** (2.16)	3.01** (2.01)
B_FemaleP	1.06 (0.70)	−0.45 (−0.30)	1.03 (0.44)	−0.45 (−0.19)
CEO_Duality	−0.91** (−2.15)	−0.57 (−1.39)	−0.91 (−1.53)	−0.57 (−1.01)
CEO_Tenure	0.34*** (5.10)	0.39*** (6.12)	0.34*** (3.99)	0.39*** (4.74)
CEO_Degree	0.86* (2.06)	1.18*** (3.23)	0.87* (1.96)	1.18* (2.00)
SOE	1.57*** (3.56)	1.23*** (2.80)	1.57** (2.03)	1.13* (1.76)
FOE	2.02*** (3.32)	2.50*** (4.18)	2.25*** (5.44)	2.24*** (3.19)
F_Size	108.60*** (32.56)	117.76*** (38.74)	108.59*** (17.51)	117.75*** (19.74)
F_Age	−0.04*** (−2.95)	−0.12*** (−3.71)	−0.11* (−1.90)	−0.12** (−2.07)
F_GrowOpp	−0.89*** (−3.93)	−1.12*** (−4.29)	−1.74*** (−2.98)	−1.12*** (−2.58)
TobinQ	0.38*** (7.57)	0.06*** (7.64)	0.38** (2.36)	0.05*** (3.70)
F_Growth	0.14 (1.11)	0.13 (1.21)	0.14 (1.41)	0.14 (1.56)
F_Leverage	2.01*** (−6.46)	−2.33*** (−7.92)	−2.12*** (−2.67)	−2.33*** (−2.52)
Cross_Listed	2.36*** (2.95)	3.14*** (3.94)	2.41** (2.24)	3.14*** (2.90)
D_Region	1.60*** (4.07)	1.98*** (5.22)	2.57*** (4.27)	2.86*** (4.63)
Constant	−345.24*** (−31.43)	−325.94*** (−32.41)	−332.61*** (−10.05)	−334.75*** (−10.07)
Year dummies	Yes	Yes	Yes	Yes
Industry dummies	Yes	Yes	Yes	Yes
Adjusted-R^2^	0.240	0.246	0.243	0.248

*T-statistics are documented in parentheses*.

***, **, * Significant at 1, 5, and 10%, respectively.

*See Table A1 in Supplementary Material for the definition of variables and Equations (3), (4) for the models' details*.

Chief executive officer (CEO) tournament incentives are linked to CSRP positively, which is consistent with H1. Our theory (that there is a substantial link between CEO tournament incentives and CEO pay) has been proved, as CEO Incentive coefficients (CEO-Incentives) are significant and have a *p*-value of 0.01. The results also support the tournament theory, which notes that if a CEO's compensation is different from that of other executives, antagonism between them will grow, resulting in increased firm output because the rewards motivate CEOs to spend more on CSR, which helps to raise the company's market profile. The findings align with Hu et al. (2013), who found a connection between CEO rewards and organizational success. Both versions featured year effects and two-digit industry codes.

Furthermore, *B_Share, B_Ind*, and *B_Size* remain important in board structure variables. The B_Size coefficient is important but negative, indicating that larger boards invest less in CSR (Garcia-Sanchez et al., 2014). This supports the theory that large boards may agonize over the lack of unity, agency dispute, and leisurely policymaking (Rao et al., 2012), and thus may be less interested in disclosing CSR-related details.

The coefficients of *B_Ind* and *B_Share*, on the other hand, remain positive and meaningful, indicating that companies with board independence and board members who own stock in their companies promote CSR investment. Previous research has shown that businesses with a high level of board independence are more likely to participate in CSR (Harjoto and Jo, 2011). However, in the models listed in Table 5, the coefficient of *CEO_Tenure* remains important and optimistic, implying that CEOs with longer tenure invest more in CSR. According to the career horizon theory, the CEO's passion for CSR investment grows as their service period increases (Chen et al., 2011). As a result, CEO tenure has a major impact on CSR efficiency. In all models listed in Table 5, the coefficient of *CEO_Degree* remains positive and important, implying that CEO education aids in improving a firm's CSP (Fernández-Gago et al., 2018).

In the models mentioned in Table 1, the coefficient of SOE remains large, implying that SOEs are more socially conscious than other firms (e.g., Khan et al., 2019). At the 1% mark, the coefficient of FOEs remains significant, indicating that firms with foreign investors/owners support more investment in CSP; as a result, firm CSP increases more in FOEs (McGuinness et al., 2017).

Moreover, F_Size and Tobin Q remain positive and highly important among the firms' economic control variables in all models listed in Table 1. The F_Age coefficient is negative, indicating that younger companies prefer social activities more than older companies, which is t with our assumptions and previous research (Marquis and Qian, 2014). Similarly, the F_GrowthOpp coefficient remains negative and important, indicating that businesses with growth opportunities are more socially conscious than other firms, possibly to improve their corporate image. The Tobin Q and F_Size coefficients are important, indicating that larger and more profitable businesses spend more on CSR than smaller businesses (Fernández-Gago et al., 2018).

The coefficient of F_Leverage is negatively significant in all models reported in Table 1. This relationship is consistent with the literature (Ali et al., 2019). The coefficient of Cross_Listed and D_Region is positively significant, suggesting cross-listed organizations and organizations traced in the more-developed canton tends to invest more in CSP because of more regulations and better corporate governance (Ali et al., 2019).

### CEO Tournament Incentives and CSRP in SOEs vs. Non-SOEs

To test the study's H2 that predicts that CEO tournament incentives' incremental effect on CSRP is more keenly recognized in state-owned organizations than their counterparts. We estimate Equations (3)–(5) for subsamples of SOEs vs. non-SOEs and the interaction effect of SOEs, respectively. Table 2 shows the effects of CEO Tournament incentives and CSRP in subsample SOEs vs. non-SOEs, using two different CEO Tournament incentive metrics (i.e., CEO Pay Gap and CEO Pay Gap Ratio). Model 1 in Table 2 boosts the regression upshots for CSP on CEO_Pay Gap in SOEs subsample. The coefficient of CEO_Pay Gap is 3.73, with a *t*-value of 10.71 indicating the CEO Tournament incentives' incremental impact on CSP is positively significant in subsample SOEs. Model 2 in Table 2 reports the same results with an alternative measure of CEO tournament incentive (i.e., CEO_Pay Gap Ratio). The coefficient of CEO_Pay Gap Ratio is 0.72 and significant at a 1% level in SOEs subsample. The results are consistent with our conjecture.

**Table 2:** CEO tournament incentive and corporate social responsibility performance (sub-sample SOEs vs. non-SOEs) and interaction effect.

	**SOE**		**Non-SOE**		**Interaction effect**	
	**Model 1**	**Model 2**	**Model 3**	**Model 4**	**Model 5**	**Model 6**
CEO_PayGap	3.73*** (10.71)	–	1.93*** (8.25)	–	1.19*** (7.30)	–
CEO_PayGapRatio	–	0.72*** (3.12)	–	0.64*** (3.63)	–	1.78*** (6.82)
CEO_PayGap × SOE	–	–	–	–	0.21*** (4.75)	–
CEO_PayGapRatio × SOE	–	–	–	–	–	0.53** (2.83)
B_Size	−0.19** (−2.05)	−0.15** (−2.01)	−0.10* (−1.06)	−0.13* (−1.71)	−0.07 (−0.74)	−0.04 (−0.37)
B_Ind	1.58 (0.39)	2.63 (0.68)	1.18 (0.45)	1.67 (0.58)	3.34 (1.09)	3.79 (1.24)
B_Share	−30.46** (−2.01)	−13.18 (−0.88)	4.63*** (3.97)	4.37*** (3.83)	6.31*** (4.64)	5.46*** (4.04)
B_FemaleP	−1.57 (−0.59)	−2.57 (−0.94)	1.59 (0.92)	1.64 (0.97)	−0.84 (−0.42)	−1.39 (−0.70)
CEO_Duality	0.73 (0.83)	1.06 (1.25)	−1.39*** (−3.11)	−1.14*** (−2.65)	−1.08* (−2.18)	−1.19* (−2.40)
CEO_Tenure	0.42*** (3.99)	0.59*** (5.85)	0.24*** (2.90)	0.26*** (3.27)	0.30*** (3.12)	0.29*** (3.46)
CEO_Degree	1.60** (2.67)	1.89*** (3.23)	1.16*** (4.13)	1.21*** (4.38)	1.31*** (4.82)	1.40*** (5.17)
F_Size	134.4*** (23.92)	153.2*** (29.05)	91.80*** (22.14)	99.65*** (26.33)	120.9*** (26.74)	118.4*** (26.71)
F_Age	−0.17*** (−2.94)	−0.20*** (−3.59)	0.10** (2.08)	0.09** (2.01)	−0.15*** (−3.42)	−0.12** (−2.90)
F_GrowOpp	−0.74*** (−2.83)	−1.11*** (−3.65)	−1.68*** (−4.19)	−2.09*** (−5.39)	−1.06*** (−3.18)	−1.22*** (−3.70)
TobinQ	−0.23 (−1.52)	−0.22 (−1.48)	0.11 (0.95)	0.23 (0.33)	0.79*** (7.24)	0.83*** (7.62)
F_Growth	1.40*** (7.26)	1.52*** (7.91)	0.21*** (4.38)	0.04*** (5.27)	0.12*** (3.96)	0.14** (2.86)
F_Leverage	−11.1*** (−11.07)	−12.6*** (−11.05)	−4.37*** (−6.77)	−4.23*** (−6.90)	−5.52*** (−7.97)	−5.78*** (−8.22)
Constant	−432.6*** (−23.12)	−456.2*** (−20.44)	−290.7*** (−17.99)	−304.8*** (−19.74)	−369.1*** (−28.29)	−363.1*** (−28.23)
Year dummies	Yes	Yes	Yes	Yes	Yes	Yes
Industry dummies	Yes	Yes	Yes	Yes	Yes	Yes
Adjusted-R^2^	0.285	0.267	0.207	0.201	0.288	0.286
Chi^2^ = 17.24, þ-value = 0.0000					–	–

*T-statistics are documented in parentheses*.

***, **, * Significant at 1, 5, and 10%, respectively.

*See Table A1 in Supplementary Material for the definition of variables and Equations (3)–(5) for the models' details*.

Model 3 in Table 2 reports regression fallouts of CSP on CEO_Pay Gap in a sub-sample of non-SOEs. The constant of CEO_Pay Gap is 1.93, with a *t*-value of 8.25, indicating the CEO Tournament incentives' incremental impact on CSP is positively significant in subsample non-SOEs. Model 4 in Table 2 reports the same results with an alternative measure of CEO tournament incentive (i.e., CEO_Pay Gap Ratio). The coefficient of CEO_Pay Gap Ratio is 0.64 and significant at level 0.01 in the sub-sample of the non-SOEs. These results are consistent with our conjecture. These results suggest that CEOs' tournament incentives in non-SOEs also lead to improved CSRP.

We applied a seemingly unrelated regression (SUR) method to compare the beta values. The chi2 coefficient is 17.24, with a *p*-value of 0 that confirms H2, which predicts that CEO tournament incentives incremental effect on CSP is highly accepted in SOEs than their counterparts.

Model 5 in Table 2 presents the regression of CSRP on CEO tournament incentive (i.e., CEO_Pay Gap) and the interaction effect of SOEs; the coefficient of CEO_Pay Gap is 1.19, and CEO_Pay Gap × SOE is 0.21 with *t*-values 7.3 and 4.75, respectively. The outcomes are in line with our view that CEO tournament incentives' incremental effect on CSRP is highly accepted in state enterprises than their counterparts. Model 6 of Table 2 states similar results with alternative CEO tournament incentive measurements (i.e., CEO_Pay Gap Ratio). The coefficient of CEO_Pay Gap Ratio is 1.78, and the coefficient of interaction between CEO_Pay Gap Ratio × SOE is 0.53, both significant at the 1 and 5% levels, respectively. These results validate our findings and support H2.

### CEO Tournament Incentives and CSRP in FOEs vs. Non-FOEs

To measure H3, FOEs are more accepting of the incremental impact of CEO tournament rewards on CSRP than their counterparts. We estimate Equations (5)–(7) for subsamples of FOEs vs. non-FOEs and the interaction effect of FOEs, respectively. The results of the interaction effect of FOEs are also reported in Table 3. Model 1 in Table 3 reports the results for the regression of CSRP on CEO_Pay Gap in FOEs subsample. The coefficient of CEO_Pay Gap is 1.21 and significant at the 5% level indicating the CEO Tournament incentives' incremental impact on CSP is positively significant in subsample FOEs. Model 2 in Table 3 reports the same results with an alternative measure of CEO tournament incentive (i.e., CEO_Pay Gap Ratio). The coefficient of CEO_Pay Gap Ratio is 0.67 and significant at the 1% level in FOEs subsample. The results are consistent with our conjecture.

**Table 3:** CEO tournament incentive and corporate social responsibility performance (sub-sample FOEs vs. non-FOEs) and interaction effect.

	**FOE**		**Non-FOE**		**Interaction effect**	
	**Model 1**	**Model 2**	**Model 3**	**Model 4**	**Model 5**	**Model 6**
CEO_PayGap	1.21** (2.91)	–	0.65*** (5.87)	–	1.65*** (9.19)	–
CEO_PayGapRatio	–	0.67*** (2.61)	–	1.24*** (2.35)	–	0.87** (2.15)
CEO_PayGap × FOE	–	–	–	–	0.12*** (2.97)	–
CEO_PayGapRatio × FOE	–	–	–	–	–	1.64*** (3.08)
B_Size	0.25 (1.13)	0.41** (1.97)	0.24*** (2.48)	0.36*** (2.75)	0.18* (1.91)	0.31*** (2.52)
B_Ind	−14.99** (−2.18)	−19.2*** (−2.84)	2.19 (1.00)	10.81*** (2.89)	−2.52 (−0.90)	8.00*** (2.23)
B_Share	9.99*** (2.72)	7.59** (2.17)	4.34*** (4.70)	2.46* (1.71)	4.56*** (5.06)	2.72* (1.95)
B_FemaleP	−5.49 (−1.18)	−0.69 (−0.41)	−0.39 (−0.28)	−0.07 (0.08)	−1.21 (−0.90)	−0.17 (−0.22)
CEO_Duality	−1.98 (−1.54)	−1.63 (−1.33)	−0.76** (−2.16)	−0.76* (−1.73)	−0.86*** (−2.64)	−0.82* (−192)
CEO_Tenure	0.34* (1.95)	0.41*** (2.87)	0.26 (4.95)	0.28*** (4.10)	0.28*** (5.55)	0.33*** (4.91)
CEO_Degree	−2.09** (−2.05)	−1.23 (−1.42)	0.74*** (2.65)	1.50*** (3.94)	0.96*** (5.20)	1.21*** (3.28)
F_Size	128.0*** (13.08)	141.2*** (15.50)	94.2*** (28.30)	7.93*** (35.08)	93.90*** (30.42)	8.08*** (38.23)
F_Age	0.18 (1.48)	0.13 (1.14)	−0.11*** (−3.17)	−0.08** (−2.07)	−0.13*** (−4.87)	−0.06 (−1.56)
F_GrowOpp	−2.05*** (−2.98)	−1.79*** (−3.34)	−1.66*** (−7.53)	−3.42*** (11.72)	−1.84*** (−8.89)	−3.54*** (−12.98)
TobinQ	0.71** (2.31)	0.58** (2.16)	0.58*** (7.59)	0.05*** (7.32)	0.67*** (9.00)	0.05*** (7.83)
F_Growth	−0.68** (−2.34)	−0.43 (−1.34)	−0.87*** (−3.80)	−0.49** (−2.11)	−0.89*** (−3.84)	−0.45* (−1.94)
F_Leverage	−14.5*** (−4.15)	−15.9*** (−5.45)	−3.29*** (−7.03)	−3.80*** (−6.81)	−4.03*** (−8.71)	−4.23*** (−7.57)
Constant	−156.3*** (−11.87)	−150.7*** (−13.84)	−112.9*** (−25.78)	−150.6*** (−26.88)	−119.9*** (−29.08)	−154.1*** (−29.29)
Year dummies	Yes	Yes	Yes	Yes	Yes	Yes
Industry dummies	Yes	Yes	Yes	Yes	Yes	Yes
Adjusted-*R*^2^	0.308		0.302	0.301	0.306	0.291
Chi^2^ = 37.35, þ-value = 0.0000					–	–

*T-statistics are documented in parentheses*.

***, **, * Significant at 1, 5, and 10%, respectively.

*See Table A1 in Supplementary Material for the definition of variables and Equations (5)–(7) for the models' details*.

Model 3 in Table 3 states the regression outcomes of CSRP on CEO_Pay Gap in the non-FOEs subsample. The coefficient of CEO_Pay Gap is 0.65, with a *t*-value of 2.91 indicating that the CEO Tournament incentives' incremental impact on CSP is positively significant in subsample non-FOEs. Model 4 in Table 3 reports the same results with an alternative measure of CEO tournament incentive (i.e., CEO_Pay Gap Ratio). The coefficient of CEO_Pay Gap Ratio is 1.24, significant at level 0.01 in the non-FOEs subsample. The results are consistent with our conjecture. These results suggest that CEOs' tournament incentives in non-FOEs also lead to improved CSP.

We applied a SUR method to compare the beta values model 1 and Model 3. The chi2 coefficient is 37.35, with a *p*-value of 0 confirming that CEO tournament incentives' incremental effect on CSP is more accepted in FOEs than their counterparts.

Model 5 in Table 3 presents regression of CSRP on CEO tournament incentive (i.e., CEO_Pay Gap) and interaction effect of FOEs; the coefficient of CEO_Pay Gap is 1.65, and CEO_Pay Gap × FOE is 0.12, with *t*-values of 9.19 and 2.97, respectively. The outcomes align with our assumption that the incremental effect of tournament incentives on CSP is more recognized in foreign-owned companies than their counterparts. Model 6 of Table 3 shows similar results with alternative CEO tournament incentive measurements (i.e., CEO_Pay Gap Ratio). The coefficient of CEO_Pay Gap Ratio is 0.87, and the coefficient of interaction between CEO_Pay Gap Ratio × FOE is 1.64, both significant at the 5 and 1% levels, respectively. These results validate our findings and support H3.

### CEO Tournament Incentives and CSRP in Cross-Listed vs. Non-cross-Listed Firms

Examining H4 of the study that predicts CEO tournament incentives' incremental effect on CSRP is more recognized in cross-listed companies than their counterparts. We calculate Equations (3), (4), and (7) for non-cross-listed and cross-listed firms and the interaction effect of cross-listed companies for subsamples of non-cross-listed and cross-listed companies, respectively. The results of the cross-listed interaction effect are also reported in Table 4. Model 1 in Table 4 shows the regression upshots of CSP on CEO_Pay Gap in the cross-listed subsample. The coefficient of CEO_Pay Gap is 5.90 and significant at the 1% level, indicating the CEO Tournament incentives' incremental impact on CSP is positively significant in subsample Cross-Listed. Model 2 in Table 4 reports the same results with an alternative measure of CEO tournament incentive (i.e., CEO_Pay Gap Ratio). The coefficient of CEO_Pay Gap Ratio is 3.59 and significant at the 1% level in the Cross-Listed subsample. The results are consistent with our conjecture. Results show the upshots of CSRP on CEO_PayGap in the non-cross-listed subsample. The coefficient of CEO_Pay Gap is 1.44, with a *t*-value of 5.43 indicating that the CEO Tournament incentives' incremental impact on CSP is positively significant in subsample non-cross-listed. Model 2 in Table 4 reports the same results with an alternative measure of CEO tournament incentive (i.e., CEO_Pay Gap Ratio). The coefficient of CEO_Pay Gap Ratio is 1.06, significant at the 5% level in the non-cross-listed subsample. The results are consistent with our conjecture. These results suggest that CEOs' non-cross-listed tournament incentives also lead to improved CSP.

**Table 4:** CEO tournament incentive and corporate social responsibility performance (sub-sample cross-listed vs. non-cross-listed) and interaction effect.

	**Cross-listed**		**Non-cross-listed**		**Interaction effect**	
	**Model 1**	**Model 2**	**Model 3**	**Model 4**	**Model 5**	**Model 6**
CEO_PayGap	5.90*** (3.90)	–	1.44*** (5.43)	–	1.57*** (5.94)	–
CEO_PayGapRatio	–	3.59*** (2.92)	–	1.06** (2.04)	–	0.97*** (2.90)
CEO_PayGap × Cross-Listed	–	–	–	–	0.37*** (4.65)	–
CEO_PayGapRatio × Cross-Listed	–	–	–	–	–	2.40*** (3.43)
B_Size	−0.34 (−0.43)	−0.03 (−0.02)	0.26* (1.77)	0.25* (1.91)	0.24* (1.65)	0.27** (2.17)
B_Ind	2.34 (1.20)	1.12 (1.21)	2.65 (0.84)	4.98* (1.95)	4.32 (1.03)	7.18** (2.00)
B_Share	−4.83*** (−3.63)	−3.56** (−2.39)	5.22*** (3.97)	4.53*** (3.49)	5.54*** (4.15)	4.79*** (3.64)
B_FemaleP	6.39 (1.17)	7.75* (1.66)	−0.19 (−0.09)	−0.85 (−0.44)	0.83 (0.42)	−0.18 (−0.23)
CEO_Duality	−7.13** (−2.07)	−1.69 (−0.62)	−1.22** (−2.45)	−0.89** (−2.08)	−1.42*** (−2.87)	−0.86** (−2.02)
CEO_Tenure	0.57* (1.65)	0.57** (2.01)	0.28*** (3.44)	0.30** (2.70)	0.29*** (3.73)	0.27*** (3.21)
CEO_Degree	0.14 (0.06)	1.27 (0.60)	1.59*** (5.75)	0.90** (2.41)	0.87** (2.09)	1.14*** (3.11)
F_Size	143.6*** (7.94)	177.2*** (9.90)	117.9*** (22.91)	127.9*** (26.62)	118.6*** (24.38)	130.7*** (28.94)
F_Age	−0.98** (−2.48)	−1.20** (−2.87)	−0.08** (−1.96)	0.11** (2.21)	−0.11** (−2.50)	−0.08** (−2.27)
F_GrowOpp	−7.28*** (−5.61)	−6.58*** (−5.58)	−2.53*** (−7.46)	−2.97*** (−10.46)	−1.38*** (−3.79)	−3.05*** (−12.92)
TobinQ	0.98*** (2.53)	0.49** (2.84)	0.83*** (6.92)	0.89*** (7.62)	0.70*** (6.28)	0.05*** (7.89)
F_Growth	−3.63* (−1.66)	−0.51 (−1.12)	−0.39 (−1.09)	−0.29 (−1.02)	0.02 (0.18)	−0.47** (−2.02)
F_Leverage	−2.58*** (−2.47)	−4.13*** (3.33)	−8.85*** (−10.12)	−8.04*** (−10.81)	−5.09*** (−7.39)	−4.34*** (−7.79)
Constant	−233.9*** (−7.35)	−216.6*** (−9.00)	−181.0*** (−27.16)	−153.0*** (−28.11)	−179.1*** (−27.99)	−152.9*** (−29.16)
Year dummies	Yes	Yes	Yes	Yes	Yes	Yes
Industry dummies	Yes	Yes	Yes	Yes	Yes	Yes
Adjusted-R^2^	0.454	0.445	0.271	0.246	0.286	0.262
Chi^2^ = 25.14, þ-value = 0.0000					–	–

*T-statistics are documented in parentheses*.

***, **, * Significant at 1, 5, and 10%, respectively.

*See Table A1 in Supplementary Material for the definition of variables and Equations (3), (4), and (7) for the models' details*.

Model 5 in Table 4 presents the regression of CSP on CEO tournament incentive (i.e., CEO_Pay Gap) and interaction effect of cross-listed; the coefficient of CEO_Pay Gap is 1.57, and CEO_Pay Gap × Cross-Listed is 0.37 with *t*-values 5.94 and 4.65, respectively. The outcomes align with our assumptions that the CEO tournament incentives' incremental effect on CSP is more recognized on cross-listed companies than their counterparts. Model 5 of Table 4 states similar results with alternative CEO tournament incentive measurements (i.e., CEO_Pay Gap Ratio). The coefficient of CEO_Pay Gap Ratio is 0.97, and the coefficient of interaction between CEO_Pay Gap Ratio × is 3.43, both significant at the 1% level.

### CEO Tournament Incentives and CSRP in More-Developed Region vs. Less-Developed Region Firms

To test H6 that predicts that CEO tournament incentives' incremental effect on CSRP is highly recognized in more-developed area companies than their counterparts. We estimate Equations (1), (2), and (4), respectively, for subsamples of developed-area and less-developed areas and the interaction effect of the developed region.

Table 5 reports the results of CEO Tournament incentives and CSRP in subsample more-D_Region vs. less-D_Region with two alternative measures of CEO Tournament incentive (i.e., CEO_Pay Gap and CEO_Pay Gap Ratio). The results of the interaction effect of cross-listed are also reported in Table 5. Model 1 in Table 5 shows the regression outcomes of CSP on CEO_Pay Gap in the D_Region subsample. The coefficient of CEO_Pay Gap is 1.56 and significant at the 1% level, representing the CEO Tournament incentives' incremental impact on CSP is positively significant in subsample D_Region. Model 2 in Table 5 reports the same results with an alternative measure of CEO tournament incentive (i.e., CEO_Pay Gap Ratio). The coefficient of CEO_Pay Gap Ratio is 0.37 and significant at the 0.01 level. The outcomes are in line with our conjecture.

**Table 5:** CEO tournament incentive and corporate social responsibility performance (sub-sample more-developed-region vs. less-developed-region) and interaction effect.

	**More-D-region**		**Less-D-region**		**Interaction effect**	
	**Model 1**	**Model 2**	**Model 3**	**Model 4**	**Model 5**	**Model 6**
CEO_PayGap	1.56*** (4.75)	–	1.29*** (3.12)	–	1.39*** (4.51)	–
CEO_PayGapRatio	–	0.37*** (2.98)	–	0.23** (2.27)	–	1.04*** (6.17)
CEO_PayGap × D_Region	–	–	–	–	0.22*** (5.63)	–
CEO_PayGapRatio × D_Region	–	–	–	–	–	1.30*** (3.91)
B_Size	0.07 (0.59)	0.15 (1.24)	0.79*** (5.01)	0.88*** (4.26)	0.27* (1.89)	0.27* (1.85)
B_Ind	1.07 (0.28)	2.44 (0.65)	4.16*** (2.67)	1.11** (2.87)	1.19 (0.38)	1.99 (0.65)
B_Share	4.14*** (2.66)	3.72** (2.39)	4.09** (2.26)	4.54** (2.46)	5.82*** (4.34)	5.30*** (4.03)
B_FemaleP	−1.69* (−1.73)	−3.62* (−1.67)	3.25** (2.44)	3.45** (2.60)	0.12 (0.06)	−0.102 (−0.05)
CEO_Duality	−1.19* (−1.97)	−0.90* (−1.74)	−0.77 (−1.36)	−0.94 (−1.21)	−1.46** (−2.97)	−1.44*** (−2.93)
CEO_Tenure	0.22* (2.04)	0.32*** (3.10)	0.42*** (3.90)	0.38*** (3.51)	0.28*** (3.62)	0.28*** (3.36)
CEO_Degree	1.99* (6.01)	2.15*** (6.59)	1.12** (2.28)	1.16** (2.41)	1.62*** (2.24)	0.94** (2.24)
F_Size	104.6*** (18.21)	116.0*** (21.32)	145.2*** (17.20)	155.7*** (19.77)	115.0*** (24.21)	133.3*** (29.56)
F_Age	0.18*** (2.88)	0.16** (2.68)	−0.18*** (−3.90)	−0.06 (−0.95)	0.16*** (3.17)	0.11** (2.20)
F_GrowOpp	−1.54*** (−3.34)	−1.71*** (−3.86)	−1.56*** (−2.67)	−2.08*** (−3.71)	−1.54*** (−4.31)	−1.65*** (−4.71)
TobinQ	0.72*** (5.21)	0.84*** (6.19)	1.01*** (5.17)	1.03*** (5.31)	0.82*** (7.08)	0.83*** (7.51)
F_Growth	−0.56** (−2.12)	−0.55** (−2.10)	−0.07 (−0.11)	−0.05 (−0.10)	−0.41 (−1.15)	−0.41 (−1.23)
F_Leverage	−4.98*** (−6.46)	−4.11*** (−5.41)	−6.88*** (−6.64)	−6.95*** (−6.73)	−5.40*** (−7.89)	−5.32*** (−7.72)
Constant	−148.6*** (−21.57)	−149.6*** (−21.41)	−182.5*** (−20.80)	−188.7*** (−21.18)	−178.9*** (−28.25)	−180.3*** (−28.48)
Year dummies	Yes	Yes	Yes	Yes	Yes	Yes
Industry dummies	Yes	Yes	Yes	Yes	Yes	Yes
Adjusted-R^2^	0.330	0.266	0.302	0.290	0.289	0.287
Chi^2^ = 27.22, þ-value = 0.0000					–	–

*T-statistics are documented in parentheses*.

***, **, * Significant at 1, 5, and 10%, respectively.

*See Table A1 in Supplementary Material for the definition of variables and Equations (3), (4), and (7) for the models' details*.

Model-3 in Table 5 reports the outcomes of regression of CSP on CEO_Pay Gap in the less-D_Region subsample. The coefficient of CEO_PayGap is 1.29, with a *t*-value of 3.12, indicating the CEO Tournament incentives' incremental impact on CSPis positively significant in subsample less-D_Region. Model 4 in Table 5 reports the same results with an alternative measure of CEO tournament incentive (i.e., CEO_Pay Gap Ratio). The coefficient of CEO_Pay Gap Ratio is 0.23 significant at the 5% level in the less-D_Region subsample. The results are consistent with our conjecture. These results suggest that CEOs' tournament incentives in less-D_Region firms also lead to improved CSP.

We applied a SUR method to compare the beta values of Models 1 and 3. The chi2 coefficient is 27.22, with a *p*-value of 0 confirming H5 that predicts CEO tournament incentives' incremental effect on CSRP is highly recognized in more-developed region firms than in less-developed region firms.

Model 5 in Table 5 presents the regression of CSRP on CEO tournament incentive (i.e., CEO_PayGap) and the interaction effect of D_Region; the coefficient of CEO_Pay Gap is 1.39, and CEO_PayGap × D_Region is 0.22 with *t*-values 4.51 and 5.63, respectively. The outcomes align with our assumption that CEO tournament incentives' incremental effect on CSRP is more recognized in companies from developed regions than their counterparts. Model 6 of Table 5 shows similar results with alternative CEO tournament incentive measurements (i.e., CEO_Pay Gap Ratio). The coefficient of CEO_Pay Gap Ratio is 1.04, and the coefficient of interaction between CEO_Pay Gap Ratio × D_Region is 1.3, both significant at the 1% level. These results validate our findings and support H5.

### Results and Discussions

Overall, the empirical results show that CEO tournament incentives motivate CEOs to be socially responsible since CEO tournament incentives are positively associated with CSP after controlling for CEO characteristics, ownership, company, and board alongside year and industry effect. Our findings confirm that CEO tournament incentives' incremental effect on CSP is highly accepted in SOEs than their counterparts. Since CEOs of SOEs may be under intense pressure from the government and other pressure groups, this result suggests that when they obtain substantial tournament rewards, they are more committed to CSRP.

The results state that CEO tournament incentives' incremental effect on CSP is highly accepted in state organizations than non-SOEs. SOE executives are likely to be under more public scrutiny as compared to non-SOE executives regarding their pay structures and CSP performance (Hu et al., 2013). Our findings demonstrate that CEO tournament incentives' incremental effect on CSP is highly recognized in FOEs than non-FOEs. Foreign owners pressurize corporations to pay for performance systems to incentivize CEOs (Firth et al., 2007). Therefore, organizations with foreign investments have higher sensitivities for the pay-performance nexus, and foreign investor ownership is associated with providing stronger pay-performance incentives to CEOs (Firth et al., 2007). The results are consistent with our conjecture. These results suggest that CEOs' non-cross-listed tournament incentives also lead to improved CSP.

The results are consistent with our conjecture and prior studies. Overall findings suggest that CEOs' non-cross-listed tournament incentives also lead to improved CSP. The Chinese cross-listed firms incorporate extra incentives in their pay design (Berrone and Gomez-Mejia, 2009). Besides, litigation risk tends to increase after cross-listing (Boubakri et al., 2016). Consequently, cross-listing encourages firm executives to boost CSP through improved governance by aligning with foreign regulations and norms, improving reputation to enhance a company's plea to stakeholders and investors, overcome foreignness liability, enhance competitiveness (Jo and Harjoto, 2011) and mitigate litigation risk and regulatory burden (Boubakri et al., 2016).

Finally, the outcomes are consistent with the literature regarding the development. Conyon and He (2011) studied executive compensation and corporate governance link in Chinese firms. They found that the CEO pay-performance link is more keenly accepted in more-developed regions than in less-developed regions. Another study reported a weaker pay-performance association from the perspective of less developed areas (Firth et al., 2007).

### Endogeneity and Further Robustness Tests

We used the firm-fixed effect regression to control the influence of unidentified firm-level characteristics and address the omitted variable concern. Model 1 in Table A5 in Supplementary Material shows the results of the firm-fixed effects regression with CEO_Pay Gap. The CEO_ Pay Gap coefficient remains positive and significant at a 1% level, which suggests that with-in firm CEO tournament incentive is positively associated with CSP. Model 2 reports the firm-fixed effect regression outcomes for the impact of CEO tournament incentive on CSRP with an alternative measure of CEO tournament incentive (CEO_Pay Gap Ratio). The coefficient of CEO_Pay Gap Ratio is 0.47 (*p* < 0.05), which confirms the previous finding. The results of the firm-fixed effect regression are in line with the previous findings and support H1 that CEO Tournament incentive has a positive association with CSP. Overall, the results obtained from firm-fixed effect regression are consistent with H1 and suggest that the relationship between CEO tournament incentives and CSRP is unlikely to be driven by endogeneity due to omitted variable bias.

Previous studies have shown CEO_Pay Gap as an endogenous variable (Conyon and He, 2011). We used the accustomed endogeneity remedy to validate our findings, a two-stage least squares regression (2-SLS). We use two alternative instrumental variables (i.e., the industry average of CEO compensation and the local average pay of CEO) of CEO tournament incentives, which are likely to meet the criterion that it is correlated with the decision to pursue a CEO tournament incentive but is not correlated with CSP. The preference of 2SLS over OLS is based on endogeneity. Table A6 in Supplementary Material describes the 2SLS regression results regarding the nexus between the CEO and CSRP tournament incentives. The table reports the first and second stages of 2SLS with both instrumental variables (i.e., local average pay and industry average pay). The first stage of 2SLS reports that the instrumental variable is positively significant. The coefficient of CEO_Pay Gap in seconds is positively significant at the 1% level, which confirms our conjecture that CEO tournament incentives motivate the CEO to be more socially responsible. The result of 2SLS validates the main regression results that state after controlling for a possible problem of endogeneity, the results are consistent.

Ordinary least squares (OLS) results may be deceptive due to self-selection preferences. In other words, the physiognomies of companies with low and high CSRP might diverge, and these characteristics may lead to differences in CSRP rather than increased tournament incentives. We follow Hung et al. (2012) to address this issue in critiquing the PSM method. We use the adjacent matching method PSM, which divides firms into a treatment group (i.e., a firm with a CEO tournament incentive) and a control group (firms without tournament incentives) having similar characteristics. We employ PSM using the probit model, where CEO tournaments (i.e., CEO_Pay Gap) are the dependent variable along with all explanatory variables. We created a dummy variable, CEO_Pay Gap, which equals 1 if the CEO_Pay Gap is higher than the sample's median, and 0 otherwise. We matched the companies grounded on entirely control variables of this study. The results of the PSM method are reported in Table A7a in Supplementary Material, and the second stage of PSM results are reported in Table A7b in Supplementary Material.

Table A7a in Supplementary Material reports the results of the first stage of the PSM model with two alternative measures of CEO tournament incentives (i.e., CEO_Pay Gap and CEO_Pay Gap Ratio). Table A7b in Supplementary Material reports the PSM results for stage two with two alternative measures of CEO tournament incentives (i.e., CEO_Pay Gap and CEO_Pay Gap Ratio). The ATT value is significant with a T-stat of 8.73 in the first model treated with CEO_Pay Gap. Similar results can be observed with an alternative measure of CEO tournament incentives. The ATT value in the model treated with CEO_Pay Gap Ratio is 2.52. The results are consistent with our conjecture that the CEO tournament incentive has a positive and significant relationship with CSP. The results of PSM are consistent with the initial results, which validate our findings.

## Conclusions and Policy Implementations

The current research explores whether tournament rewards encourage CEOs to increase their investment in CSR activities. This study employs recent data from all A-share listed enterprises in the Chinese stock markets to investigate it. After controlling for factors such as ownership and board structure and economic variables regarding an organization, findings suggest that CEOs invest more in CSR projects when they receive comparatively better incentives. Our results are in line with tournament theory, which suggests that bonuses and prizes drive managers. Growth-inducing salary rewards allow executives to compete with one another, allowing the company to flourish financially and socially.

This study attempts to broaden insights into CSRP and the effect of sub-national institutional contingencies. The outcomes of this study reveal that sub-national institutional contingencies (i.e., firms in less-developed vs. more-developed regions, non-cross-listed vs. cross-listed companies, non-FOEs vs. FOEs, non-SOEs vs. SOEs) positively affect CSRP. The fallouts of this research divulge that CSP in firms in more-develop areas, cross-listed companies, FOEs, and SOEs are higher than their counterparts. The explanations for the upper CSRP in cross-listed companies or companies headquartered in more-developed regions, or FOES or SOEs, are shareholder protection, state pressure, proper security supervision, information asymmetry, CSR regulations, media coverage, and legal standards. The study results reveal that sub-national institutional contingencies affect the association between CEO tournament incentives and CSRP. Still, this relationship is more keenly felt in SOEs, FOEs, cross-listed firms, and firms in more-developed regions.

Although this study has achieved the anticipated research goals and found some essential research conclusions, the research has a few limitations that offer new and interesting opportunities for future research. As China's market diverges from developed countries, there might be an issue of generalizability of the results, whereas our sample relies on listed companies in an emerging economy. Thus, the role of CEO tournament incentives in CSRP may not be suitable for unlisted firms or firms in developed markets. Consequently, future researchers are encouraged to test the hypotheses in other developing and developed countries to enhance and endorse the generalizability of the results. Second, although a series of tests were performed to tackle the endogeneity, we used only one proxy for CSRP. Future studies may use other variables for CSRP to establish a causal link. Fourth, in our analysis, the findings concentrate on China's institutional climate, an increasingly changing economy, and different from developed countries in numerous ways. Moreover, all sub-national institutional contingencies (i.e., firms in less-developed vs. more-developed regions, non-cross-listed vs. cross-listed companies, non-FOEs vs. FOEs, non-SOEs vs. SOEs) are derived from a single country. Future studies should consider how this is affected by other sub-national institutions (e.g., family businesses vs. non-family firms and semi-governmental firms). Moreover, future research is advised to extend this study in multinational settings and thereby enhance the generalizability of the findings.

## Data Availability Statement

The data analyzed in this study is subject to the following licenses/restrictions: data is available on the CSMAR website. Requests to access these datasets should be directed to http://cndata1.csmar.com.

## Author Contributions

MK: conceptualization, methodology, formal analysis, review, and editing. SA: data curation, formal analysis, writing, review, and editing. RZ: investigation, writing, review, and editing. CH: resources and supervision. MN: review, editing, and conceptualization. All authors contributed to the article and approved the submitted version.

## Funding

This research was supported by the National Social Science Fund of China (21BGL047).

## Conflict of Interest

The authors declare that the research was conducted in the absence of any commercial or financial relationships that could be construed as a potential conflict of interest.

## Publisher's Note

All claims expressed in this article are solely those of the authors and do not necessarily represent those of their affiliated organizations, or those of the publisher, the editors and the reviewers. Any product that may be evaluated in this article, or claim that may be made by its manufacturer, is not guaranteed or endorsed by the publisher.

## References

[B1] Ali S. Zhang J. Usman M. Khan F. U. Ikram A. Anwar B. (2019). Sub-national institutional contingencies and corporate social responsibility performance: evidence from China. Sustainability 11, 5478. 10.3390/su11195478

[B2] Barnea A. Rubin A. (2010). Corporate social responsibility as a conflict between shareholders. J. Bus. Ethics 97, 71–86. 10.1007/s10551-010-0496-z12125883

[B3] Bebchuk L. A. Fried J. Walker D. (2002). Managerial Power and Rent Extraction in the Design of Executive Compensation. Cambridge, MA: National Bureau of Economic Research.

[B4] Becker B. E. Huselid M. A. (1992). The incentive effects of tournament compensation systems. Admin. Sci. Q. 336–350. 10.2307/2393228

[B5] Berrone P. Gomez-Mejia L. R. (2009). Environmental performance and executive compensation: an integrated agency-institutional perspective. Acad. Manag. J. 52, 103–126. 10.5465/amj.2009.36461950

[B6] Bondy K. Moon J. Matten D. (2012). An institution of corporate social responsibility (CSR) in multi-national corporations (MNCs): form and implications. J. Bus. Ethics 111, 281–299. 10.1007/s10551-012-1208-7

[B7] Boubakri N. El Ghoul S. Wang H. Guedhami O. Kwok C. C. (2016). Cross-listing and corporate social responsibility. J. Corp. Fin. 41, 123–138. 10.1016/j.jcorpfin.2016.08.008

[B8] Bruton G. D. Ahlstrom D. Chen J. (2021). China has emerged as an aspirant economy. Asia Pac. J. Manag. 38, 1–15. 10.1007/s10490-018-9638-0

[B9] Cai Y. Jo H. Pan C. (2011). Vice or virtue? The impact of corporate social responsibility on executive compensation. J. Bus. Ethics 104, 159–173. 10.1007/s10551-011-0909-7

[B10] Campbell J. L.. (2007). Why would corporations behave in socially responsible ways? An institutional theory of corporate social responsibility. Acad. Manag. Rev. 32, 946–967. 10.5465/amr.2007.25275684

[B11] Cassimon D. Engelen P. J. Van Liedekerke L. (2016). When do firms invest in corporate social responsibility? A real option framework. J. Bus. Ethics 137, 15–29. 10.1007/s10551-015-2539-y

[B12] Chan C. M. Makino S. Isobe T. (2010). Does subnational region matter? Foreign affiliate performance in the United States and China. Strateg. Manag. J. 31, 1226–1243. 10.1002/smj.854

[B13] Chen J. Ezzamel M. Cai Z. (2011). Managerial power theory, tournament theory and executive pay in China. J. Corp. Fin. 17, 1176–1199. 10.1016/j.jcorpfin.2011.04.008

[B14] Cheung Y. L. Kong D. Tan W. Wang W. (2015). Being good when being international in an emerging economy: the case of China. J. Bus. Ethics 130, 805–817. 10.1007/s10551-014-2268-7

[B15] Connelly B. L. Tihanyi L. Crook T. R. Gangloff K. A. (2014). Tournament theory: thirty years of contests and competitions. J. Manag. 40, 16–47. 10.1177/0149206313498902

[B16] Conyon M. J. He L. (2011). Executive compensation and corporate governance in China. J. Corp. Fin. 17, 1158–1175. 10.1016/j.jcorpfin.2011.04.006

[B17] Conyon M. J. He L. (2014). CEO turnover in China: the role of market-based and accounting performance measures. Eur. J. Fin. 20, 657–680. 10.1080/1351847X.2012.676559

[B18] Conyon M. J. Peck S. I. Sadler G. V. (2001). Corporate tournaments and executive compensation: evidence from the UK. Strateg. Manag. J. 22, 805–815. 10.1002/smj.169

[B19] Cordeiro J. J. He L. Conyon M. Shaw T. S. (2013). Informativeness of performance measures and Chinese executive compensation. Asia Pac. J. Manag. 30, 1031–1058. 10.1007/s10490-013-9353-9

[B20] David P. Bloom M. Hillman A. J. (2007). Investor activism, managerial responsiveness and corporate social performance. Strateg. Manag. J. 28, 91–100. 10.1002/smj.571

[B21] David P. Yoshikawa T. Chari M. D. Rasheed A. A. (2006). Strategic investments in Japanese corporations: do foreign portfolio owners foster underinvestment or appropriate investment? Strateg. Manag. J. 27, 591–600. 10.1002/smj.523

[B22] Davis G. F.. (2005). New directions in corporate governance. Annu. Rev. Sociol. 31, 143–162. 10.1146/annurev.soc.31.041304.122249

[B23] Del Bosco B. Misani N. (2016). The effect of cross-listing on the environmental, social and governance performance of firms. J. World Bus. 51, 977–990. 10.1016/j.jwb.2016.08.002

[B24] Doidge C. Karolyi G. A. Stulz R. M. (2004). Why are foreign firms listed in the US worth more? J. Fin. Econ. 71, 205–238. 10.1016/S0304-405X(03)00183-1

[B25] Elkins H.. (2018). Measuring compensation system structure: the interrelation between equitable pay and firm performance. 10.2139/ssrn.3198893

[B26] Elsayed N. Elbardan H. (2018). Investigating the associations between executive compensation and firm performance: agency theory or tournament theory. J. Appl. Account. Res. 19, 245–270. 10.1108/JAAR-03-2015-002726857680

[B27] Fan G. Wang X. Zhang L. W. Zhu H. (2003). Marketization index for China's provinces. Econ. Res. J. 3, 9–18.

[B28] Fan J. P. Wong T. J. Zhang T. (2007). Politically connected CEOs, corporate governance and post-IPO performance of China's newly partially privatized firms. J. Fin. Econ. 84, 330–357. 10.1016/j.jfineco.2006.03.008

[B29] Fernández-Gago R. Cabeza-García L. Nieto M. (2018). Independent directors' background and CSR disclosure. Corp. Soc. Responsib. Environ. Manag. 25, 991–1001. 10.1002/csr.1515

[B30] Firth M. Fung P. M. Rui O. M. (2006). Corporate performance and CEO compensation in China. J. Corp. Fin. 12, 693–714. 10.1016/j.jcorpfin.2005.03.00233551900

[B31] Firth M. Fung P. M. Rui O. M. (2007). How ownership and corporate governance influence chief executive pay in China's listed firms. J. Bus. Res. 60, 776–785. 10.1016/j.jbusres.2007.01.014

[B32] Galaskiewicz J.. (1997). An urban grants economy revisited: corporate charitable contributions in the Twin Cities, 1979–81, 1987–89. Admin. Sci. Q. 1997, 445–471. 10.2307/2393734

[B33] Garcia-Sanchez I. M. Cuadrado-Ballesteros B. Sepulveda C. (2014). Does media pressure moderate CSR disclosures by external directors? Manag. Decis. 52, 1014–1045. 10.1108/MD-09-2013-0446

[B34] Gnyawali D. R. Offstein E. H. Lau R. S. (2008). The impact of the CEO pay gap on firm competitive behavior. Group Organ. Manag. 33, 453–484. 10.1177/1059601108321637

[B35] Goel A. M. Thakor A. V. (2008). Overconfidence, CEO selection and corporate governance. J. Fin. 63, 2737–2784. 10.1111/j.1540-6261.2008.01412.x

[B36] Gomez-Mejia L. R.. (1994). Executive compensation: a reassessment and a future research agenda. Res. Person. Human Resourc. Manag. 12, 161–222.

[B37] Grofčíková J.. (2020). Impact of selected determinants of corporate governance on financial performance of companies. Econ. Manag. Spectrum 14, 12–24. 10.26552/ems.2020.2.12-23

[B38] Guariglia A. Yang J. (2016). A balancing act: managing financial constraints and agency costs to minimize investment inefficiency in the Chinese market. J. Corp. Fin. 36, 111–130. 10.1016/j.jcorpfin.2015.10.006

[B39] Hail L. Leuz C. (2009). Cost of capital effects and changes in growth expectations around US cross-listings. J. Fin. Econ. 93, 428–454. 10.1016/j.jfineco.2008.09.006

[B40] Hambrick D. C.. (2007). Upper echelons theory: an update. Acad. Manag. Rev. 32, 334–343. 10.5465/amr.2007.24345254

[B41] Hambrick D. C. Mason P. A. (1984). Upper echelons: the organization as a reflection of its top managers. Acad. Manag. Rev. 9, 193–206. 10.5465/amr.1984.4277628

[B42] Hannan R. L. Krishnan R. Newman A. H. (2008). The effects of disseminating relative performance feedback in tournament and individual performance compensation plans. Account. Rev. 83, 893–913. 10.2308/accr.2008.83.4.893

[B43] Harjoto M. A. Jo H. (2011). Corporate governance and CSR nexus. J. Bus. Ethics 100, 45–67. 10.1007/s10551-011-0772-632642899

[B44] He L. Fang J. (2016). Subnational institutional contingencies and executive pay dispersion. Asia Pacific J. Manag. 33, 371–410. 10.1007/s10490-015-9429-9

[B45] Hofstede G.. (2001). Culture's Consequences: Comparing Values, Behaviors, Institutions and Organizations Across Nations. London: Sage publications.

[B46] Hong B. Li Z. Minor D. (2016). Corporate governance and executive compensation for corporate social responsibility. J. Bus. Ethics 136, 199–213. 10.1007/s10551-015-2962-0

[B47] Hu F. Pan X. Tian G. (2013). Does CEO pay dispersion matter in an emerging market? Evidence from China's listed firms. Pac. Basin Fin. J. 24, 235–255. 10.1016/j.pacfin.2013.07.003

[B48] Hung M. Wong T. J. Zhang T. (2012). Political considerations in the decision of Chinese SOEs to list in Hong Kong. J. Account. Econ. 53, 435–449. 10.1016/j.jacceco.2011.10.001

[B49] Jensen M. C. Meckling W. H. (1976). Theory of the firm: managerial behavior, agency costs and ownership structure. J. Fin. Econ. 3, 305–360. 10.1016/0304-405X(76)90026-X

[B50] Jian M. Lee K. W. (2015). CEO compensation and corporate social responsibility. J. Multinatl. Fin. Manag. 29, 46–65. 10.1016/j.mulfin.2014.11.00410276817

[B51] Jiraporn P. Chintrakarn P. (2013). How do powerful CEOs view corporate social responsibility (CSR)? An empirical note. Econ. Lett. 119, 344–347. 10.1016/j.econlet.2013.03.026

[B52] Jo H. Harjoto M. A. (2011). Corporate governance and firm value: the impact of corporate social responsibility. J. Bus. Ethics 103, 351–383. 10.1007/s10551-011-0869-y34566768

[B53] Khan F. U. Zhang J. Usman M. Badulescu A. Sial M. S. (2019). Ownership reduction in state-owned enterprises and corporate social responsibility: perspective from secondary privatization in China. Sustainability 11, 1008. 10.3390/su11041008

[B54] Khan M. K. He Y. Akram U. Sarwar S. (2017). Financing and monitoring in an emerging economy: can investment efficiency be increased? China Econ. Rev. 45, 62–77. 10.1016/j.chieco.2017.05.012

[B55] Khan M. K. Zahid R. M. Saleem A. Sági J. (2021). Board composition and social and environmental accountability: a dynamic model analysis of chinese firms. Sustainability 13, 10662. 10.3390/su131910662

[B56] Kini O. Williams R. (2012). Tournament incentives, firm risk and corporate policies. J. Fin. Econ. 103, 350–376. 10.1016/j.jfineco.2011.09.005

[B57] Krüger P.. (2015). Corporate goodness and shareholder wealth. J. Fin. Econ. 115, 304–329. 10.1016/j.jfineco.2014.09.008

[B58] Lang M. H. Lins K. V. Miller D. P. (2003). ADRs, analysts and accuracy: does cross listing in the United States improve a firm's information environment and increase market value? J. Account. Res. 41, 317–345. 10.1111/1475-679X.00106

[B59] Lau C. M. Lu Y. Liang Q. (2016). Corporate social responsibility in China: A corporate governance approach. J. Bus. Ethics. 136, 73–87. 10.1007/s10551-014-2513-0

[B60] Lazear E. P. Rosen S. (1981). Rank-order tournaments as optimum labor contracts. J. Polit. Econ. 89, 841–864. 10.1086/261010

[B61] Li D. Lin H. Yang Y. W. (2016). Does the stakeholders–corporate social responsibility (CSR) relationship exist in emerging countries? Evidence from China. Soc. Responsib. J. 12, 147–166. 10.1108/SRJ-01-2015-0018

[B62] Lu J. Ren L. Zhang C. Qiao J. Kovacova M. Streimikis J. (2020). Assessment of corporate social responsibility and its impacts on corporate reputation of companies in selected Balkan Countries former Yugoslavia States. Technol. Econ. Dev. Econ. 26, 504–524. 10.3846/tede.2020.12069

[B63] Malkawi E. Khayrullina M. (2021). Digital human skills form the corporate economy and business development. Econ. Manag. Spectr. 15, 64–75. 10.26552/ems.2021.1.64-74

[B64] Marquis C. Qian C. (2014). Corporate social responsibility reporting in China: symbol or substance? Organ. Sci. 25, 127–148. 10.1287/orsc.2013.0837

[B65] Matten D. Moon J. (2008). “Implicit” and “explicit” CSR: a conceptual framework for a comparative understanding of corporate social responsibility. Acad. Manag. Rev. 33, 404–424. 10.5465/amr.2008.31193458

[B66] McCarthy S. Oliver B. Song S. (2017). Corporate social responsibility and CEO confidence. J. Bank. Fin. 75, 280–291. 10.1016/j.jbankfin.2016.11.024

[B67] McGuinness P. B. Vieito J. P. Wang M. (2017). The role of board gender and foreign ownership in the CSR performance of Chinese listed firms. J. Corp. Fin. 42, 75–99. 10.1016/j.jcorpfin.2016.11.001

[B68] McGuire J. Oehmichen J. Wolff M. Hilgers R. (2019). Do contracts make them care? The impact of CEO compensation design on corporate social performance. J. Bus. Ethics 157, 375–390. 10.1007/s10551-017-3601-8

[B69] Muller A. Kolk A. (2010). Extrinsic and intrinsic drivers of corporate social performance: Evidence from foreign and domestic firms in Mexico. J. Manag. Stud. 47, 1–26. 10.1111/j.1467-6486.2009.00855.x

[B70] Musacchio A. Lazzarini S. G. Aguilera R. V. (2015). New varieties of state capitalism: strategic and governance implications. Acad. Manag. Perspect. 29, 115–131. 10.5465/amp.2013.0094

[B71] Ntim C. G. Soobaroyen T. (2013). Corporate governance and performance in socially responsible corporations: New empirical insights from a Neo-Institutional framework. Corp. Govern. 21, 468–494. 10.1111/corg.12026

[B72] O'Reilly C. A III Main B. G. Crystal G. S. (1988). CEO compensation as tournament and social comparison: A tale of two theories. Admin. Sci. Q. 1988, 257–274. 10.2307/2393058

[B73] Petrenko O. V. Aime F. Ridge J. Hill A. (2016). Corporate social responsibility or CEO narcissism? CSR motivations and organizational performance. Strateg. Manag. J. 37, 262–279. 10.1002/smj.2348

[B74] Rao K. K. Tilt C. A. Lester L. H. (2012). Corporate Governance and Environmental Reporting: An Australian Study. Corporate Governance

[B75] Reese Jr W. A. Weisbach M. S. (2002). Protection of minority shareholder interests, cross-listings in the United States and subsequent equity offerings. J. Fin. Econ. 66, 65–104. 10.1016/S0304-405X(02)00151-4

[B76] Rosen S.. (1986). Prizes and incentives in elimination tournaments. Am. Econ. Rev. 76, 701–715.

[B77] Shi W. Sun S. L. Peng M. W. (2012). Sub-national institutional contingencies, network positions and IJV partner selection. J. Manag. Stud. 49, 1221–1245. 10.1111/j.1467-6486.2012.01058.x

[B78] Smith K. Sofianos G. (1997). The Impact of an NYSE Listing on the Global Trading of Non-US Stocks. Vol. 97. Citeseer.

[B79] Southam C. Sapp S. (2010). Compensation across executive labor markets: what can we learn from cross-listed firms? J. Int. Bus. Stud. 41, 70–87. 10.1057/jibs.2009.34

[B80] Vo T. T. N. Canil J. M. (2019). CEO pay disparity: efficient contracting or managerial power? J. Corp. Fin. 54, 168–190. 10.1016/j.jcorpfin.2016.10.002

[B81] Waldman D. A. Sully de Luque M. Washburn N. House R. J. Adetoun B. Barrasa A.. (2006). Cultural and leadership predictors of corporate social responsibility values of top management: a GLOBE study of 15 countries. J. Int. Bus. Stud. 37, 823–837. 10.1057/palgrave.jibs.8400230

[B82] Wu W. Johan S. A. Rui O. M. (2016). Institutional investors, political connections and the incidence of regulatory enforcement against corporate fraud. J. Bus. Ethics 134, 709–726. 10.1007/s10551-014-2392-4

[B83] Xu E. Yang H. Quan J. M. Lu Y. (2015). Organizational slack and corporate social performance: Empirical evidence from China's public firms. Asia Pac. J. Manag. 32, 181–198. 10.1007/s10490-014-9401-0

[B84] Yoshikawa T. Phan P. H. David P. (2005). The impact of ownership structure on wage intensity in Japanese corporations. J. Manag. 31, 278–300. 10.1177/0149206304271766

[B85] Yoshikawa T. Rasheed A. A. Del Brio E. B. (2010). The impact of firm strategy and foreign ownership on executive bonus compensation in Japanese firms. J. Bus. Res. 63, 1254–1260. 10.1016/j.jbusres.2010.06.012

[B86] Zahid R. A. Simga-Mugan C. (2019). An analysis of IFRS and SME-IFRS adoption determinants: a worldwide study. Emerg. Markets Fin. Trade 55, 391–408. 10.1080/1540496X.2018.1500890

[B87] Zhang F. Zhang H. Brown D. H. Yin X. (2021). Innovation and performance of manufacturing firms in aspirant markets: an institutional environment approach. Asia Pac. J. Manag. 2021, 1–48. 10.1007/s10490-021-09790-w

[B88] Zhao X. Zhou G. Rezaee Z. (2021). Tournament incentives and corporate social responsibility performance. J. Account. Audit. Fin. 2021:0148558X211022946. 10.1177/0148558X211022946

[B89] Zheng H. Zhang Y. (2016). Do SOEs outperform private enterprises in CSR? Evidence from China. Chin. Manag. Stud. 10, 435–457. 10.1108/CMS-10-2015-0225

[B90] Zheng S. Kahn M. E. Sun W. Luo D. (2014). Incentives for China's urban mayors to mitigate pollution externalities: The role of the central government and public environmentalism. Reg. Sci. Urban Econ. 47, 61–71. 10.1016/j.regsciurbeco.2013.09.003

