# Supplementary material for: Does Whipping Tournament Incentives Spur CSR Performance? An Empirical Evidence From Chinese Sub-national Institutional Contingencies
Source: Front Psychol. 2022 Feb 25;13:841163. doi: 10.3389/fpsyg.2022.841163 (PMC8913534; doi:10.3389/fpsyg.2022.841163)
Supplement: Supplementary file 1 [file Table_1.DOCX]

Supplementary Material

**
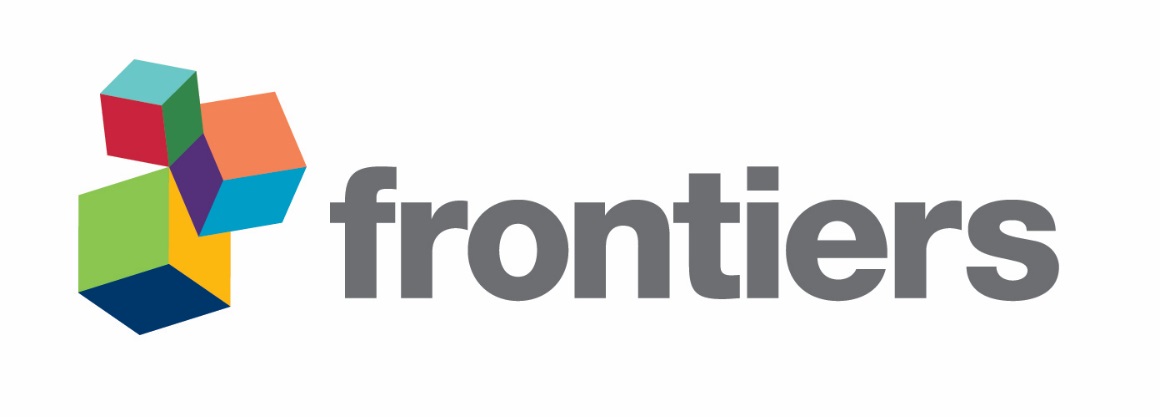
**

**Supplementary Material**

**Appendix:**

| **Table: A1 Description of variables** | |
| --- | --- |
| Variables | Description |
| CSR_Rating | CSR rating score is weighted average ranging from 0 to 100 issued by Rankins (RKS). |
| CEO_PayGap | The logarithm of total CEO compensation minus the average pay of other executives. |
| CEO_PayGapRatio | The ratio of CEO and other executives’ average compensation. |
| SOE | A dummy variable, which equals 1 if the local or central government is the dominant owner and otherwise 0. |
| FOE | A dummy variable that equals 1 if the foreign investors owned shares in a firm and 0 otherwise |
| Cross_Listed | A dummy variable that equals 1 if the firm is cross-listed Hong Kong Stock Exchange and 0 if it is non-cross-listed. |
| D_Region | A dummy variable that equals 1 if the firm’s head office is located in more developed regions of China, and 0 otherwise. |
| B_Size | The number of directors on the board. |
| B_Ind | The proportion of outside directors on the board. |
| B_Share | The percentage of shares held by a firm’s board of directors. |
| B_ FemaleP | The proportion of female directors on the board. |
| CEO_Duality | A dummy variable that equals one if the CEO is working as chairperson and otherwise 0. |
| CEO_Tenure | CEO tenure is the number of years since the CEO was appointed as the CEO. |
| CEO_Degree | A dummy variable which equals 1 if the CEO has at least a bachelor degree 0 otherwise |
| F_Size | The log of total sales. |
| F_Age | The number of years the firm has listed on the stock exchange. |
| F_GrowOpp | Equals to the book to market ratio. A ratio of a publically-traded company’s book value to its market value. |
| TobinQ | The industry-adjusted Tobin’s Q ratio. Calculated as the market value of assets over the book value of assets |
| F_Growth | Change in a firm’s total assets. |
| F_ Leverage | The ratio of total debt to the total asset. |

| **Table A2: Descriptive Statistics** | | | | |
| --- | --- | --- | --- | --- |
| Variable | Mean | Std. Dev. | Min | Max |
| CSP | 27.61 | 14.29 | 0.00 | 89.29 |
| CEO_PayGap | 407000 | 549000 | -915000 | 15,000,000 |
| CEO_PayGapRatio | 2.59 | 1.20 | 0.00 | 18.46 |
| B_Size | 10.26 | 2.63 | 5.00 | 27.00 |
| B_Ind | 0.38 | 0.07 | 0.20 | 0.80 |
| B_Share | 0.10 | 0.18 | 0.00 | 0.89 |
| B_FemaleP | 0.13 | 0.12 | 0.00 | 0.83 |
| CEO_Duality | 0.24 | 0.42 | 0.00 | 1.00 |
| CEO_Tenure | 3.44 | 2.93 | 0.00 | 19 |
| CEO_Degree | 0.93 | 0.25 | 0.00 | 1 |
| SOE | 0.46 | 0.49 | 0.00 | 1.00 |
| FOE | 0.05 | 0.23 | 0.00 | 1 |
| F_Size | 21.98 | 1.25 | 13.76 | 28.00 |
| F_ Age | 9.10 | 6.22 | 0.00 | 28 |
| F_GrowOpp | 0.98 | 0.99 | 0.00 | 18.45 |
| TobinQ | 2.76 | 27.93 | 0.08 | 2354.73 |
| F_Growth | 0.83 | 1.91 | -12.81 | 64.70 |
| F_Leverage | 0.45 | 0.36 | 0.00 | 16.55 |
| Cross_Listed | 0.06 | 0.24 | 0.00 | 1 |
| D_Region | 0.64 | 0.48 | 0.00 | 1 |
| *Table 1, shows a detail explanation of variables* | | | | |

| Table A4 Pairwise Correlation Matrix | | | | | | | | | | | | | | | | | | | | | |  |
| --- | --- | --- | --- | --- | --- | --- | --- | --- | --- | --- | --- | --- | --- | --- | --- | --- | --- | --- | --- | --- | --- | --- |
| Variables | 1 | | 2 | | 3 | | 4 | | 5 | | 6 | | 7 | | 8 | | 9 | | 10 | | | |
| 1.CSP | 1.00 | | ---- | | ---- | | ---- | | ---- | | ---- | | ---- | | ---- | | ---- | | ---- | | | |
| 2.CEO_PayGap | 0.25* | | 1.00 | | ---- | | ---- | | ---- | | ---- | | ---- | | ---- | | ---- | | ---- | | | |
| 3.CEO_PayGapRatio | 0.04* | | 0.58* | | 1.00 | | ---- | | ---- | | ---- | | ---- | | ---- | | ---- | | ---- | | | |
| 4.B_Size | 0.06* | | 0.12* | | 0.14* | | 1.00 | | ---- | | ---- | | ---- | | ---- | | ---- | | ---- | | | |
| 5.B_Ind | 0.01 | | 0.02* | | -0.01 | | -0.07* | | 1.00 | | ---- | | ---- | | ---- | | ---- | | ---- | | | |
| 6.B_Share | -0.09* | | -0.14* | | -0.11* | | -0.17* | | 0.11* | | 1.00 | | ---- | | ---- | | ---- | | ---- | | | |
| 7.B_FemaleP | -0.07* | | -0.04* | | 0.02* | | -0.07* | | 0.04* | | 0.14* | | 1.00 | | ---- | | ---- | | ---- | | | |
| 8.CEO_Duality | -0.08* | | 0.02 | | 0.03* | | -0.11* | | 0.07* | | 0.26* | | 0.09* | | 1.00 | | ---- | | ---- | | | |
| 9.CEO_Tenure | 0.03* | | 0.12* | | 0.07* | | -0.03* | | 0.11* | | 0.05* | | 0.04* | | 0.09* | | 1.00 | | ---- | | | |
| 10.CEO_Degree | 0.14* | | 0.13* | | 0.03* | | 0.09* | | 0.01 | | -0.14* | | -0.08* | | -0.03* | | 0.02 | | 1.00 | | | |
| 11.SOE | 0.16* | | 0.07* | | 0.03* | | 0.22* | | -0.10* | | -0.49* | | -0.19* | | -0.29* | | -0.10* | | 0.19* | | | |
| 12.FOE | 0.11* | | 0.08* | | -0.01 | | 0.04* | | 0.02* | | -0.06* | | -0.01 | | -0.03* | | 0.011 | | 0.03* | | | |
| 13.F_Size | 0.36* | | 0.30* | | -0.011 | | 0.22* | | 0.02* | | -0.27* | | -0.15* | | -0.16* | | 0.03* | | 0.14* | | | |
| 14.F_Age | 0.05* | | 0.13* | | 0.09* | | 0.14* | | -0.06* | | -0.55* | | -0.07* | | -0.22* | | 0.02* | | 0.11* | | | |
| 15.F_GrowOpp | 0.18* | | 0.09* | | -0.03* | | 0.12* | | -0.00 | | -0.24* | | -0.09* | | -0.13* | | -0.02* | | 0.11* | | | |
| 16.TobinQ | -0.03* | | -0.03* | | -0.02 | | -0.03* | | 0.01 | | 0.01 | | -0.00 | | 0.00 | | -0.01 | | -0.01 | | | |
| 17.F_Growth | 0.06* | | 0.02* | | 0.03* | | 0.06* | | -0.01 | | 0.06* | | 0.01 | | 0.05* | | -0.07* | | -0.01 | | | |
| 18.F_Leverage | -0.05* | | 0.05* | | 0.02 | | 0.09* | | -0.01 | | -0.23* | | -0.05* | | -0.09* | | -0.05* | | 0.05* | | | |
| 19.Cross_Listed | 0.07* | | 0.08* | | 0.01 | | 0.02 | | -0.00 | | -0.01 | | 0.00 | | -0.013 | | 0.06* | | 0.02 | | | |
| 20.D_Region | 0.07* | | 0.15* | | 0.02* | | -0.06* | | 0.00 | | 0.14* | | 0.05* | | 0.09* | | 0.06* | | 0.01 | | | |
| *CONTINUE TABLE A4* | | | | | | | | | | | | | | | | | | | | | |  |
| Variables | | | 11 | | 12 | | 13 | | 14 | | 15 | | 16 | | 17 | | 18 | | 19 | | 20 |  |
| 1.CSP | | | ---- | | ---- | | ---- | | ---- | | ---- | | ---- | | ---- | | ---- | | ---- | | ---- |  |
| 2.CEO_PayGap | | | ---- | | ---- | | ---- | | ---- | | ---- | | ---- | | ---- | | ---- | | ---- | | ---- |  |
| 3.CEO_PayGapRatio | | | ---- | | ---- | | ---- | | ---- | | ---- | | ---- | | ---- | | ---- | | ---- | | ---- |  |
| 4.B_Size | | | ---- | | ---- | | ---- | | ---- | | ---- | | ---- | | ---- | | ---- | | ---- | | ---- |  |
| 5.B_Ind | | | ---- | | ---- | | ---- | | ---- | | ---- | | ---- | | ---- | | ---- | | ---- | | ---- |  |
| 6.B_Share | | | ---- | | ---- | | ---- | | ---- | | ---- | | ---- | | ---- | | ---- | | ---- | | ---- |  |
| 7.B_FemaleP | | | ---- | | ---- | | ---- | | ---- | | ---- | | ---- | | ---- | | ---- | | ---- | | ---- |  |
| 8.CEO_Duality | | | ---- | | ---- | | ---- | | ---- | | ---- | | ---- | | ---- | | ---- | | ---- | | ---- |  |
| 9.CEO_Tenure | | | ---- | | ---- | | ---- | | ---- | | ---- | | ---- | | ---- | | ---- | | ---- | | ---- |  |
| 10.CEO_Degree | | | ---- | | ---- | | ---- | | ---- | | ---- | | ---- | | ---- | | ---- | | ---- | | ---- |  |
| 11.SOE | | | 1.00 | | ---- | | ---- | | ---- | | ---- | | ---- | | ---- | | ---- | | ---- | | ---- |  |
| 12.FOE | | | 0.08* | | 1.00 | | ---- | | ---- | | ---- | | ---- | | ---- | | ---- | | ---- | | ---- |  |
| 13.F_Size | | | 0.35* | | 0.17* | | 1.00 | | ---- | | ---- | | ---- | | ---- | | ---- | | ---- | | ---- |  |
| 14.F_Age | | | 0.44* | | 0.04* | | 0.21* | | 1.00 | | ---- | | ---- | | ---- | | ---- | | ---- | | ---- |  |
| 15.F_GrowOpp | | | 0.31* | | 0.05* | | 0.51* | | 0.23* | | 1.00 | | ---- | | ---- | | ---- | | ---- | | ---- |  |
| 16.TobinQ | | | -0.04* | | -0.01 | | -0.13* | | 0.00 | | -0.06* | | 1.00 | | ---- | | ---- | | ---- | | ---- |  |
| 17.F_Growth | | | -0.08* | | -0.01 | | 0.01 | | -0.05* | | -0.03* | | 0.09* | | 1.00 | | ---- | | ---- | | ---- |  |
| 18.F_Leverage | | | 0.19* | | -0.01 | | 0.21* | | 0.27* | | 0.32* | | 0.37* | | 0.02 | | 1.00 | | ---- | | ---- |  |
| 19.Cross_Listed | | | 0.03* | | 0.06* | | 0.06* | | 0.05* | | 0.06* | | -0.00 | | -0.00 | | 0.02* | | 1.00 | | ---- |  |
| 20.D_Region | | | 0.06* | | -0.01 | | -0.00 | | -0.10* | | -0.05* | | 0.02 | | -0.00 | | -0.06* | | 0.02 | | 1.00 |  |
| *Table shows detailed explanation of variables; * shows 0.05 level significance* | | | | | | | | | | | | | | | | | | | | | |  |

| **Table A5: Robustness test of CEO Tournament Incentive on corporate social responsibility performance: Firm fixed effects** | | |
| --- | --- | --- |
|  | Model 1 | Model 2 |
| CEO_PayGap | 0.88***  (3.52) | ---- |
| CEO_PayGapRatio | ---- | 0.47**  (2.78) |
| B_Size | -0.19***  (-2.92) | -0.21**  (-2.47) |
| B_Ind | 2.18**  (2.47) | -5.68**  ( -2.18) |
| B_Share | 0.66  (0.20) | 2.89***  (3.04) |
| B_FemaleP | 4.61*  (1.72) | 5.15**  (1.97) |
| CEO_ Duality | 0.59  (0.10) | -0.29  (-0.37) |
| CEO_ Tenure | 0.24***  (3.13) | 0.21***  (3.35) |
| CEO_Degree | -0.47  (-0.63) | -0.20  (-0.35) |
| SOE | -2.47*  (-1.78) | -2.23*  (-1.67) |
| FOE | 0.18  (0.33) | 0.33  (0.62) |
| F_Size | 77.98***  (11.19) | 73.42***  (11.43) |
| F_ Age | -1.85***  (-16.87) | -1.57***  (-14.82) |
| F_GrowOpp | 2.21***  (7.94) | 1.38***  (4.77) |
| TobinQ | 0.23***  (3.43) | 0.02*  (1.86) |
| F_Growth | 0.17***  (2.87) | -0.04  (-0.54) |
| F_ Leverage | -3.68***  (-3.98) | -2.64***  (-3.07) |
| Constant | -60.34***  (-4.50) | -57.78***  (-5.64) |
| Year Dummies | YES | YES |
| Industry Dummies | YES | YES |
| Adjusted-R^2^ | 0.06 | 0.06 |
| Hausman Chi^2^ | 409.86  (0.000) | 371.99  (0.000) |
| T-statistics are documented in parentheses. ***, **, *, significant at 1, 5, and 10%, respectively. See Table A1 for the definition of variables. | | |

| **Table A6: Robustness test of CEO Tournament Incentive on Corporate Social Responsibility Performance (2SLS)** | | | | | | | |
| --- | --- | --- | --- | --- | --- | --- | --- |
|  | First-Stage | | Second-Stage | | First-Stage | | Second-Stage |
| Local Average Pay | 4.53***  (36.66) | | ---- | | ---- | | ---- |
| Industry Average Pay | ---- | | ---- | | 3.73***  (33.05) | | ---- |
| CEO_PayGap | ---- | | 4.15***  (10.17) | | ---- | | 4.41***  (9.88) |
| B_Size | -0.05***  (-5.18) | | 0.38**  (2.57) | | -0.05***  (-4.68) | | 0.39**  (2.62) |
| B_Ind | -0.57**  (-1.99) | | 9.46**  (2.21) | | -0.64**  (-2.21) | | 9.74**  (2.26) |
| B_Share | 1.43  (1.02) | | 1.34  (0.64) | | 1.76  (1.24) | | 1.30***  (5.11) |
| B_FemaleP | 0.06  (1.03) | | -0.11  (-0.12) | | -0.44***  (-4.08) | | -0.15  (-0.16) |
| CEO_ Duality | 0.19***  (5.73) | | -1.84***  (-3.61) | | 0.7**  (2.45) | | -1.89***  (-3.69) |
| CEO_ Tenure | 0.03***  (4.71) | | 0.19**  (2.44) | | 0.03***  (6.77) | | 0.19**  (2.34) |
| CEO_Degree | 0.02  (0.60) | | 0.62  (1.45) | | 0.10***  (6.74) | | 0.99***  (2.70) |
| SOE | -0.29***  (-8.59) | | 3.36***  (6.28) | | -0.32***  (-9.04) | | 3.45***  (5.66) |
| FOE | -0.07*  (-1.71) | | 1.25**  (2.17) | | 0.16***  (3.54) | | 1.88**  (2.01) |
| F_Size | 0.27**  (2.81) | | 38.21***  (25.37) | | 0.56***  (5.66) | | 37.75***  (24.47) |
| F_ Age | 0.02***  (7.09) | | -0.17***  (-3.90) | | 0.02***  (3.72) | | 0.16***  (2.72) |
| F_GrowOpp | -0.01  (-0.38) | | -2.96***  (-9.00) | | -0.02  (0.51) | | -2.93***  (-8.88) |
| TobinQ | 0.01**  (2.27) | | 0.70***  (8.06) | | 0.02***  (2.81) | | 0.69***  (7.86) |
| F_Growth | 0.01  (0.16) | | -0.32  (-0.87) | | 0.01  (0.02) | | 0.30**  (2.20) |
| F_ Leverage | -0.04  (-0.88) | | -5.34***  (-7.47) | | -0.03  (-0.69) | | -3.65***  (-4.86) |
| Cross_Listed | -4.10***  (-8.03) | | 4.93***  (2.68) | | 1.87***  (4.76) | | 4.45**  (1.97) |
| D_Region | 0.01  (0.40) | | 2.05***  (4.63) | | 0.26***  (9.79) | | 2.00***  (4.49) |
| Constant | 9.52***  (20.30) | | -197.65***  (-29.31) | | 10.69***  (66.84) | | -198.61***  (-29.19) |
| Year & Industry Dummies | YES | | YES | | YES | | YES |
| Adjusted-R^2^ | 0.272 | | 0.267 | | 0.248 | | 0.260 |
| Wald Chi^2^ | ---- | | 2753.38*** | | ---- | | 2724.23*** |
| T-statistics are documented in parentheses. ***, **, *, significant at 1, 5, and 10%, respectively. See Table A1 for the definition of variables. | | | | | | | |
| **Table A7a: Robustness test of CEO Tournament Incentive on Corporate Social Responsibility Performance (PSM)** | | | | | | | |
|  | | PSM-OLS | | First-Stage | | First-Stage  (Alt. Measure) | |
| CEO_PayGap | | 2.78***  (5.95) | | ---- | | ---- | |
| B_Size | | -0.17*  (-1.87) | | -0.04***  (-5.16) | | -0.03***  (-3.58) | |
| B_Ind | | 2.29  (1.34) | | 0.24  (0.83) | | -1.22***  (-4.40) | |
| B_Share | | 2.13*  (1.65) | | -0.45***  (-4.22) | | -0.76***  (-6.88) | |
| B_FemaleP | | 1.61  (0.83) | | 0.16***  (2.68) | | -0.15**  (-2.56) | |
| CEO_ Duality | | -1.70*  (-1.75) | | 0.19***  (2.81) | | 0.16***  (4.01) | |
| CEO_ Tenure | | 0.19**  (2.45) | | 0.02***  (3.39) | | 0.02**  (2.36) | |
| CEO_Degree | | 0.09***  (3.13) | | 0.14***  (3.39) | | 0.03  (1.44) | |
| SOE | | 2.42***  (2.78) | | 0.41***  (11.35) | | 0.16***  (4.69) | |
| FOE | | 0.12**  (2.12) | | 0.19***  (3.01) | | 0.08**  (2.23) | |
| F_Size | | 78.81***  (14.30) | | 1.26***  (14.33) | | -0.41***  (-4.97) | |
| F_ Age | | -0.15**  (-2.44) | | 0.03***  (5.35) | | 0.02***  (3.20) | |
| F_GrowOpp | | 1.79***  (4.53) | | 0.04*  (1.71) | | 0.03*  (1.83) | |
| TobinQ | | 0.28*  (1.90) | | 0.04***  (5.84) | | 0.02**  (2.99) | |
| F_Growth | | 0.14  (0.36) | | 0.01  (1.08) | | -0.05**  (-1.97) | |
| F_ Leverage | | -6.23***  (-2.74) | | -0.08*  (-1.84) | | -0.04  (0.87) | |
| Cross_Listed | | 0.23**  (1.98) | | 0.34***  (3.58) | | 0.05*  (1.81) | |
| D_Region | | 0.52***  (4.15) | | 0.34***  (8.91) | | 0.03**  (2.23) | |
| Constant | | -1.32***  (-8.47) | | -5.28***  (-13.96) | | -1.23***  (-7.98) | |
| Year Dummies | | YES | | ---- | | ---- | |
| Industry Dummies | | YES | | ---- | | ---- | |
| Pseudo R^2^ | | 0.253 | | 0.07 | | 0.06 | |
| T-statistics are documented in parentheses. ***, **, *, significant at 1, 5, and 10%, respectively. See Table A1 for the definition of variables. | | | | | | | |

| **Table A7b: Robustness test of CEO Tournament incentive on corporate social responsibility performance (PSM)** | | | | | | |
| --- | --- | --- | --- | --- | --- | --- |
| Treated with CEO_PayGap | | | | | | |
| CSP | Sample | Treated | Controls | Difference | S.E. | T-stat |
|  | Unmatched | 31.5078 | 25.0606 | 6.44712 | .48986 | 13.16 |
|  | ATT | 31.5078 | 25.4222 | 6.085594 | .69723 | 8.73 |
| Treated with CEO_PayGapRatio (alternative measure) | | | | | | |
| CSP | Sample | Treated | Controls | Difference | S.E. | T-stat |
|  | Unmatched | 30.1271 | 26.8503 | 3.2767 | .34432 | 9.52 |
|  | ATT | 30.1271 | 28.9201 | 1.2070 | .47877 | 2.52 |
